# Chromatin loops associated with active genes and heterochromatin shape rice genome architecture for transcriptional regulation

**DOI:** 10.1038/s41467-019-11535-9

**Published:** 2019-08-13

**Authors:** Lun Zhao, Shuangqi Wang, Zhilin Cao, Weizhi Ouyang, Qing Zhang, Liang Xie, Ruiqin Zheng, Minrong Guo, Meng Ma, Zhe Hu, Wing-Kin Sung, Qifa Zhang, Guoliang Li, Xingwang Li

**Affiliations:** 1 National Key Laboratory of Crop Genetic Improvement, Huazhong Agricultural University, 1 Shizishan Street, Hongshan District, Wuhan, 430070 Hubei China; 2 Department of Resources and Environment, Henan University of Engineering, 1 Xianghe Road, Longhu Town, Zhengzhou, 451191 Henan China; 3 State Key Laboratory of Agricultural Microbiology, Huazhong Agricultural University, 1 Shizishan Street, Hongshan District, Wuhan, 430070 Hubei China; 4 Department of Computer Science, National University of Singapore, 13 Computing Drive, Singapore, 117417 Singapore; 5 Genome Institute of Singapore, 60 Biopolis Street, Genome, Singapore, 138672 Singapore; 6 Hubei Key Laboratory of Agricultural Bioinformatics and Hubei Engineering Technology Research Center of Agricultural Big Data, Huazhong Agricultural University, 1 Shizishan Street, Hongshan District, Wuhan, 430070 Hubei China

**Keywords:** Agricultural genetics, Plant genetics, Epigenomics

## Abstract

Insight into high-resolution three-dimensional genome organization and its effect on transcription remains largely elusive in plants. Here, using a long-read ChIA-PET approach, we map H3K4me3- and RNA polymerase II (RNAPII)-associated promoter–promoter interactions and H3K9me2-marked heterochromatin interactions at nucleotide/gene resolution in rice. The chromatin architecture is separated into different independent spatial interacting modules with distinct transcriptional potential and covers approximately 82% of the genome. Compared to inactive modules, active modules possess the majority of active loop genes with higher density and contribute to most of the transcriptional activity in rice. In addition, promoter–promoter interacting genes tend to be transcribed cooperatively. In contrast, the heterochromatin-mediated loops form relative stable structure domains in chromatin configuration. Furthermore, we examine the impact of genetic variation on chromatin interactions and transcription and identify a spatial correlation between the genetic regulation of eQTLs and e-traits. Thus, our results reveal hierarchical and modular 3D genome architecture for transcriptional regulation in rice.

## Abstract (web summary)

Three-dimensional genome organization and its effect on transcription remain elusive in rice. Here, the authors map promoter–promoter interactions and heterochromatin interactions using ChIA-PET and reveal spatial correlation between the genetic regulation of eQTLs and e-traits.

## Introduction

Revealing the structural and functional relationship of the three-dimensional (3D) genome is one of the fundamental questions in biology. Chromosome conformation capture-derived approaches, especially high-throughput chromosome conformation capture (Hi-C)^1–5^ and chromatin interaction analysis by paired-end tag sequencing (ChIA-PET)^6,7^, have characterized the hierarchical 3D genome architecture in mammals^2,8–12^. CTCF/cohesin and other DNA-binding proteins, as well as RNAs, have been identified as key factors in the spatial organization of long-range chromatin interactions^2,13–17^. Chromatin loops mediated by CTCF/cohesin and other protein factors coalesce into chromatin contact domains/topologically associating domains (TADs) and further aggregate into active and inactive compartments and chromosome territories. In addition, our and other studies revealed that CTCF and RNAPII-organized chromatin interactions provide a topological basis for transcriptional regulation^8,9,18–20^. Recent investigations have also thoroughly explored 3D genome dynamics and functions during the cell cycle, development, and signaling in multiple cells and in single cells^21–25^. Overall, the mammalian genome is organized as a complex and delicate structure, which is divided into different TAD/compartment categories with distinct epigenetic features. In addition, this organization plays a critical role in gene transcription. Similarly, 3D genome maps for *Arabidopsis*, rice, and other plants have been generated using the Hi-C approach^26–31^. However, due to the limited resolution of Hi-C^6^, comprehensive high-resolution chromatin architecture, including both active and inactive elements/compartments, and its effects on transcriptional regulation remain unclear in plants.

In this study, we present comprehensive long-range chromatin interaction maps including active *cis*-regulatory elements and heterochromatin regions using RNA polymerase II (RNAPII) and histone modifications (H3K4me3 and H3K9me2)-associated long-read ChIA-PET data in rice (*Oryza sativa*). By integrating transcriptome and epigenome data analysis, we provide details regarding 3D genome architecture and framework for transcriptional regulation. We also reveal the impact of genetic variation on chromatin interactions and transcription regulation. Furthermore, we demonstrate the spatial correlation between the genetic regulation of expression quantitative trait loci (eQTLs) and e-traits. Collectively, these results, along with recent reports on the long-range chromatin interaction maps in maize^32,33^, provide high-resolution views on 3D genome architecture and its role in transcriptional regulation in plants.

## Results

### Multiscale 3D genome maps in rice

To investigate the genome architecture organization principle and its effects on transcriptional regulation in rice, we mapped active genes related chromatin architecture by capturing RNAPII and H3K4me3-associated chromatin interactions and present heterochromatin-engaged genome architecture using H3K9me2-associated interactions in young leaves of two rice varieties, Minghui 63 (MH63) and Zhenshan 97 (ZS97). We collected ~329 million uniquely mapped paired-end tags (2 × 150 bp) from 12 ChIA-PET libraries (Supplementary Table 1). Considering the high reproducibility of biological replicates for each ChIA-PET dataset category at different resolutions (Supplementary Fig. 1), we combined biological replicate data for downstream analysis.

**Fig. 1:**
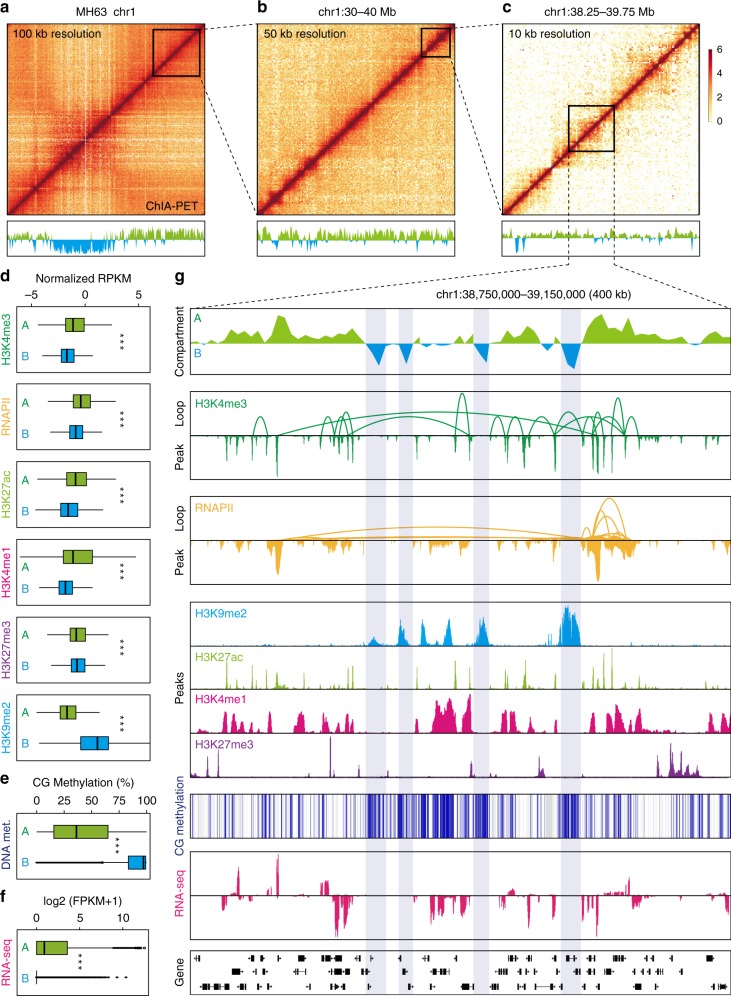
ChIA-PET data for high-resolution 3D genome mapping in rice. **a**–**c** Chromatin interaction heatmaps and A/B compartments of chromosome 1 in rice variety MH63. The upper graphs show ChIA-PET (combined RNAPII and H3K4me3 data) heatmaps at 100-kb resolution (**a**), 50-kb resolution (**b**), and 10-kb resolution (**c**). The lower graphs show the A (green histogram) and B compartments (blue histogram) represented by the first eigenvector from principal component analysis (PCA). **d**–**f** Features of epigenetic modifications and gene transcription in higher-order structures (A and B compartments). ***p < 0.001 from Wilcoxon test. Boxplots show the median, and third and first quartiles. **g** Epigenetic features of chromatin loops and binding peaks in the box region of panel **c**. The data tracks show A/B compartments, chromatin loops mediated by H3K4me3 and RNAPII, profiling of representative histone modifications and RNAPII occupancy, CG methylation level, and gene transcription. Highlighted B compartments enriched with H3K9me2 modification and CG methylation. Source data of **e**, **f** are provided as a Source data file

**Fig. 2:**
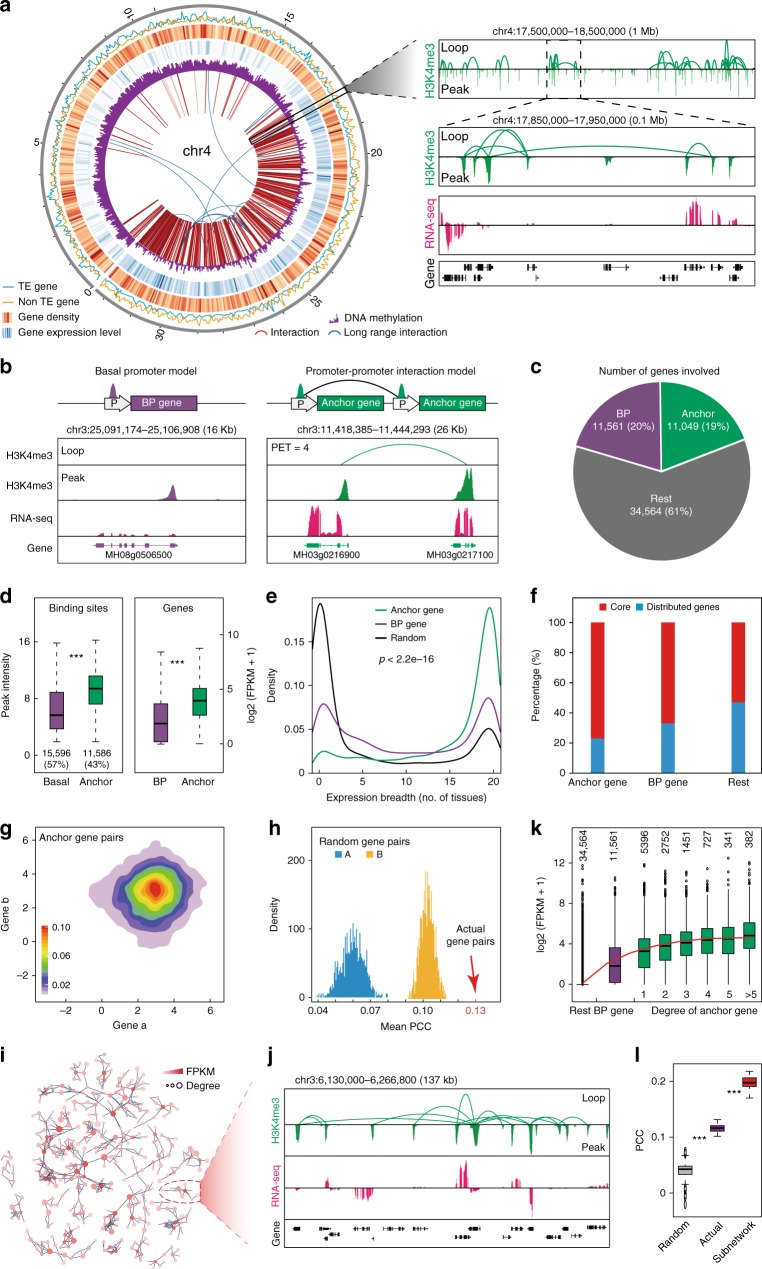
Characterization of H3K4me3-associated promoter–promoter interactions. **a** Global view of H3K4me3-associated interactions and epigenetic and transcriptional features on chr4. Zoomed-in view of detailed loop and peak distributions at the indicated regions. **b** Chromatin models. The BP model, genes with binding sites that are not involved in chromatin interactions. The PPI model, genes with anchor sites involved in interactions. P promoter; solid curve, chromatin loop. **c** Pie chart for the percentage and number of genes engaged in H3K4me3-associated chromatin models. **d** Boxplot for intensities of H3K4me3 peaks (left) and expression levels of BP and anchor genes (right). ***p < 0.001 from Wilcoxon test. **e** Expression breadth of H3K4me3 anchor and BP genes. Random genes served as control. *p* < 2.2e−16 from Kruskal–Wallis test. **f** Distribution of core and distributed genes that participated in PPI models. Core genes, present in more than 99% of 453 rice accessions; distributed genes, present in <99% of 453 rice accessions. **g** Contour plot of log-transformed FPKM values for H3K4me3 anchor gene pairs. **h** Histogram for co-expression analysis of H3K4me3 anchor gene pairs. The mean PCC of anchor gene pairs is much higher than that of both (A) randomly simulated gene pairs and (B) randomly selected H3K4me3-marked gene pairs which have the same physical distance with anchor gene pairs. **i** H3K4me3-associated PPI network from chromosome 3. The size of the circles is proportional to the degree (connection frequency) of the genes (as nodes). The redder color indicates higher gene expression. **j** A screenshot of a H3K4me3-associated cluster in dotted line box area of **h**. **k** Boxplot shows the positive relationship of the degrees of genes with transcriptional activity. Gene numbers in each category are given. **l** Co-expression analysis of genes in H3K4me3-associated subnetworks. The PCC of subnetworks are calculated. Random, randomly selected gene pairs. Actual, all H3K4me3 anchor gene pairs. ***p < 0.001 from Wilcoxon test. Boxplots in **d**, **k**, **l** show the median, and third and first quartiles. Source data of **d**, **k** are provided as a Source data file

We first examined the genome organization at the chromosome level with the combined ChIA-PET map at a low resolution (Fig. 1a–c). We observed intense chromatin interactions among the end regions of all chromosomes (Supplementary Fig. 2a), suggesting that chromosomal ends are clustered together in rice nuclei. Furthermore, principal component analysis showed that the genome was partitioned into two categories of compartments (A and B) (Supplementary Fig. 2c), with ~65% of the rice genome belonging to A compartments. Compared with B compartments, A compartments exhibited significantly higher intensities of active histone marks (H3K4me3, H3K27ac, and H3K4me1) and RNAPII, as well as significantly higher transcriptional levels. By contrast, the peak intensities of heterochromatic marks (H3K9me2 and DNA methylation) were significantly lower in A compartments than that in B compartments (Fig. 1d–f). These low-resolution results from our ChIA-PET data showed patterns similar to those from the Hi-C map in recent studies^28,30^. Strikingly, the ChIA-PET data mainly comprised the following types of information at higher resolution (100 bp level) than did the Hi-C data: the target protein-binding sites and chromatin interactions between binding sites mediated by the targeted protein (Fig. 1g; Fig. 2a). Taken together, our ChIA-PET data include not only the high-order organization of the rice genome at a low resolution but also the comprehensive pairwise high-resolution chromatin loops connecting the target protein-bound and histone modification-marked DNA elements.

### Characterization of long-range promoter–promoter loops

We then examined the features of promoter–promoter chromatin interactions with H3K4me3 ChIA-PET data. Of 27,182 H3K4me3-binding sites, ~43% served as anchors which were involved in chromatin interactions; thus, they were termed as the promoter–promoter interaction (PPI) model (Fig. 2b). We identified 11,230 high-confidence (false discovery rate (FDR < 0.05)) PPI connecting 11,049 active genes (Fig. 2c), where majority of chromatin interactions (~87%, 9714) were intrachromosomal (Supplementary Table 1) with a median loop size of ~16 kb (Supplementary Fig. 3a). Thus, different chromosomes were assigned to relatively independent specific nuclear spaces depending on the frequency of chromatin interactions (Supplementary Fig. 4). These interactions were widely distributed throughout the whole genome and were enriched in noncentromeric regions (Supplementary Fig. 5a). By contrast, the remaining H3K4me3 peaks that were not involved in interactions may reflect the basic promoter function for gene transcription and were termed as the basal promoter (BP) model. In addition, the peak intensity of anchor sites as well as the expression level of anchor genes in the PPI model tended to be higher than those in the BP model (Fig. 2d). We further found that the most of H3K27ac peaks were overlapped with H3K4me3 peaks (Supplementary Fig. 6a), and H3K27ac-marked anchor genes showed higher expression level than H3K27ac-marked basal genes (Supplementary Fig. 6b). These results suggest that PPIs are basic genome architecture units that connect active genes, which exhibit higher expression levels than do BP genes.

We further characterized the expression patterns of genes of the PPI model and the basal model. We analyzed RNA-seq data from 20 different rice tissues (Supplementary Table 2) and found that H3K4me3 anchor genes tended to be expressed in most of the examined tissues (Fig. 2e), indicating that they are housekeeping genes. By contrast, BP genes were placed in both tissue-specific and housekeeping categories (Fig. 2e). Consistently, GO analysis revealed that the anchor genes were significantly enriched in terms related to basic functional metabolic processes (Supplementary Fig. 3g). These results indicate that widely expressed genes are more likely to be involved in chromatin interactions.

We also compared the conservation of anchor and BP genes by investigating their distribution in the following gene categories obtained from pan-genome analysis:^34^ core genes (including candidate core genes) occurred in >99% of 453 rice accessions, and distributed genes occurred in <99% of rice accessions. Compared with the BP model, the PPI model contained the highest proportion of core genes and the lowest proportion of distributed genes, whereas the non-H3K4me3-marked genes included the lowest proportion of core genes and the highest proportion of distributed genes (Fig. 2f). Thus, genes associated with chromatin loops have a stronger retention capacity in different rice accessions during the process of rice genome evolution compared with BP genes and the rest genes.

### Spatially interacting genes show correlated transcription

We speculated that the spatial proximity of the anchor genes’ promoters may contribute to their cooperative transcriptional regulation. To test this hypothesis, we first investigated the expression level of anchor genes in one type of tissue. RNA-seq data showed that most paired anchor genes are highly expressed simultaneously (Fig. 2g), and further analysis indicated that the mean Pearson correlation coefficient (PCC) of anchor gene pairs in 20 different tissues (Supplementary Table 2) was far beyond that of randomly simulated gene pairs with the same physical distance (Fig. 2h), suggesting that chromatin looping contributes to the improved co-expression correlation of interacting gene pairs. We also identified 2901 (26%) anchor promoters connected with at least three interacting gene partners (Fig. 2i). These clusters formed complex architectures with an average of 49 kb genomic span, and the anchor genes with higher degrees displayed higher transcriptional activities (Fig. 2j, k). In addition, the anchor genes in the same subnetworks tended to be more co-expressed (Fig. 2l). These results indicate that widespread PPIs may play a significant role in transcription regulation.

### PPI loop regions display distinct epigenomic properties

We classified H3K4me3-associated loops into seven groups based on epigenetic modification patterns to explore their chromatin features (Supplementary Fig. 7). Notably, Group 5 (inactive loops) was covered with H3K9me2 in which genes were inactive and had larger genomic spans than did the active loops. Group 6 (inactive loops) with the largest loop span was nearly depleted of any marks and most loop genes were silenced. Despite distinct epigenetic properties as well as loop span and transcription activity of loop genes in different groups, the expression intensity of the anchor genes at each loop end showed no significant differences in all groups (Supplementary Fig. 7d). Thus, PPIs could cross genomic regions with different epigenetic properties (even heterochromatin) and clustered active genes to functional foci in specific spatial domains.

### Active chromatin loops orchestrate cooperative transcription

We then proceeded to analyze RNAPII-associated chromatin interactions. Similar to H3K4me3 anchors, 44% (8037) of RNAPII-binding sites were involved in interactions and tended to be with higher intensity than did basal sites (Supplementary Fig. 3b). In addition, RNAPII anchor genes tended to be higher transcriptional activity, expression breadth, co-expression levels, and core gene proportion than did BP genes (Supplementary Fig. 3b–f). Notably, we identified more RNAPII-associated interactions (28,213) but connected fewer active genes (7465) than those of H3K4me3 (Supplementary Table 1; Supplementary Fig. 3b). In addition, the RNAPII loops had a broader span (~21–392 kb) than did H3K4me3 loops (~11–27 kb) (Supplementary Fig. 3a).

We further investigated the effects of H3K4me3 and RNAPII-associated chromatin interactions on gene transcription. Most (~62%, 4594) RNAPII anchor genes were also involved in H3K4me3-associated genome architecture. When compared with genes anchored by only H3K4me3 or RNAPII loops, genes engaged in both H3K4me3 and RNAPII loops had the highest transcriptional activity (Supplementary Fig. 6c). Similarly, H3K4me3 BP genes had relatively higher expression levels when occupied by RNAPII (Supplementary Fig. 6d). To characterize the spatial relations between RNAPII- and H3K4me3-associated chromatin topology, we defined structural domains as chromosome interacting domains (CIDs) based on connectivity and contact frequency (see Method). Most (76%, 1692) H3K4me3-related active interacting domains (AID-related) were embedded in RNAPII-related transcriptional interacting domains (TID-related) (Supplementary Fig. 6e). These observations suggest that RNAPII- and H3K4me3-associated chromatin interactions form complex spatial transcriptional units and cooperatively orchestrate gene transcription.

### Identification of heterochromatic interacting domains

**Fig. 3:**
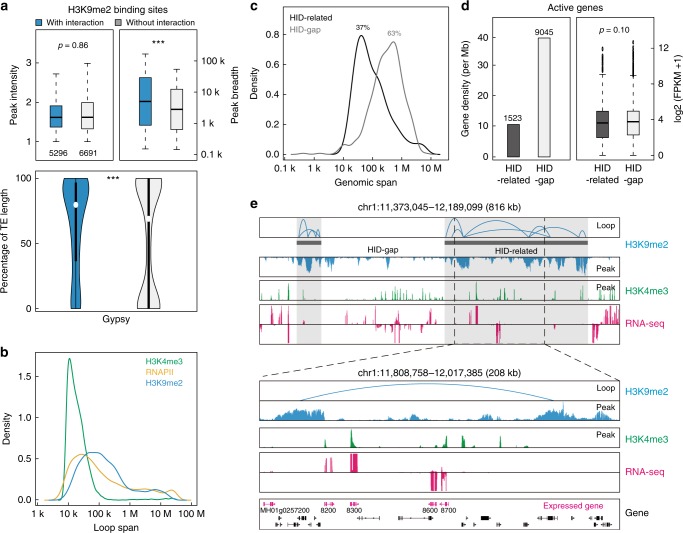
Identification of Heterochromatin-related chromatin interacting domains. **a** Boxplots for peak intensity (upper left), peak breadth (upper right), and transposable element (TE) occupancy (bottom panel) of H3K9me2-binding sites involved and not involved in interactions. ***p < 0.001 from Wilcoxon test. **b** Loop span distribution of interactions mediated by H3K9me2 (5627), H3K4me3 (9714), and RNAPII (17160). **c** Genome span distribution of HID-related and HID-gap regions. The percentages of their genome occupancy are shown above. **d** Density and transcriptional level of active genes (marked by H3K4me3 anchors) located in HID-related and HID-gap regions. *p* = 0.10 from Wilcoxon test. **e** Representative screenshot for one H3K9me2-related heterochromatic interacting domains (HID-related) showing H3K9me2 ChIA-PET data overlapped with H3K4me3 ChIP-Seq and RNA-seq data. There were a few H3K4me3 peaks and expressed genes in the HID-related segments with lower densities compared with those in HID-gap segments (the genomic regions excluding HID-related regions). A few expressed genes (magenta) marked by H3K4me3 were observed inside the H3K9me2 loop. Boxplots in **a**, **d** show the median, and third and first quartiles. Source data of **a**, **d** are provided as a source data file

H3K9me2-marked heterochromatin covered a significant portion of the rice genome—specifically, 21% of the rice genome, which prompted us to examine the 3D genome organization mediated by H3K9me2-marked heterochromatin. Through our research, we find that ~44% (5296) of H3K9me2-marked regions acted as interaction anchors that show significantly longer peak breadth but equal peak intensities compared with those of basal sites (Fig. 3a). We also observe that the majority class of transposable element (TE) involved in H3K9me2 modification was *Gypsy* retrotransposon (Supplementary Fig. 8a), and the anchor sites contained higher *Gypsy* density than did basal sites (Fig. 3a), indicating that longer H3K9me2-binding sites with higher TE density tend to be involved in chromatin interactions.

Finally, we identify 11,590 high-confidence (FDR < 0.05) H3K9me2-associated heterochromatin interaction loops, with approximate half of these loops being intrachromosomal interactions (Supplementary Table 1) enriched in the centromeric and pericentromeric regions (Supplementary Fig. 5b), and possessing the longest loop span (~39–341 kb; median width, 105 kb) compared with RNAPII and H3K4me3 loops (Fig. 3b). Strikingly, interchromosomal interactions were also enriched in the centromeric and pericentromeric regions, suggesting that clustering of centromeres is mediated by heterochromatin interactions in rice nuclei.

Many H3K9me2-marked regions involved loops interconnected with a broader span of genome segments. Based on loop connectivity and contact frequency, we identified 385 H3K9me2-related heterochromatic interacting domains (HID-related) with a genomic span from 10 kb to 8.5 Mb, accounting for 37% of the rice genome (Fig. 3c). Interestingly, despite a higher density of TE genes versus a lower density of non-TE genes and expressed genes in HID-related segments than in HID-gaps (the remaining genomic regions except HID-related segments), we found no significant difference in the expression level of the active genes between these two domains (Supplementary Fig. 8b, c; Fig. 3d, e). Together, our H3K9me2 ChIA-PET data provide a heterochromatic interactome as a basic and larger genome architecture unit with lower transcription potential, mainly due to the lower distribution of expressed genes.

### Interweaving modular CIDs form genome architecture

**Fig. 4:**
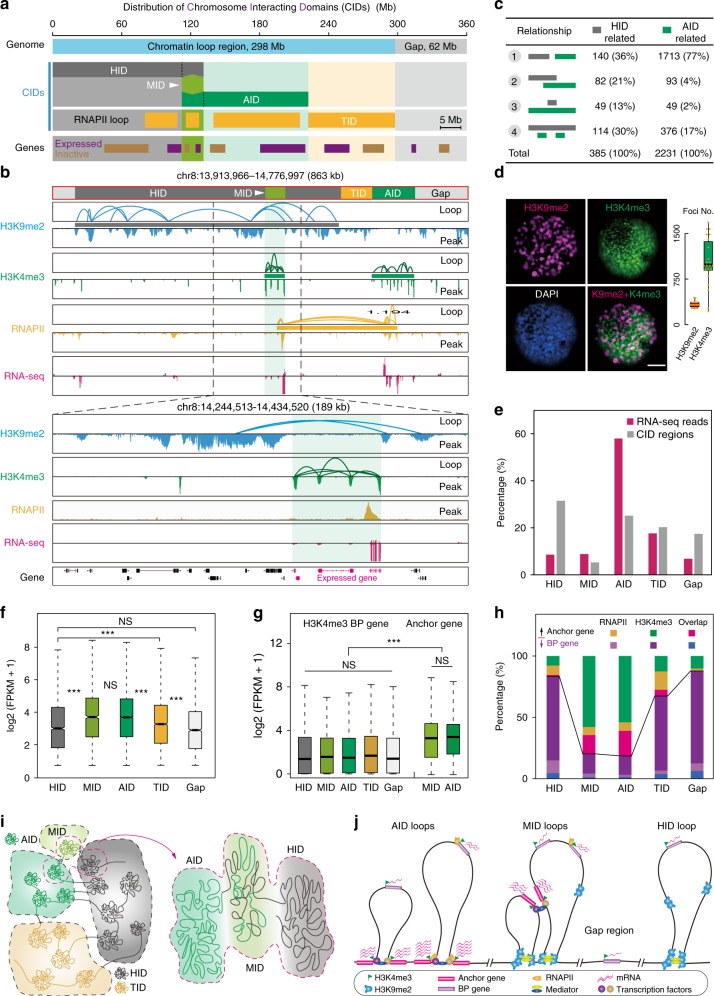
Rice chromatin organization and its transcriptional function. **a** Bar chart of genomic coverage by different chromosome interacting domains (CIDs). The majority of the rice genome is covered by CIDs (297 Mb, 82%), whereas a minor portion is gap regions not covered by chromatin interactions (63 Mb, 18%). HIDs, 114 Mb, 32%; AIDs, 91 Mb, 25%; MIDs, 19 Mb, 5%, and TIDs, 73 Mb, 20%. **b** Browser view of different CIDs showing interval arrangement. Dotted box represents a MID segment. Anchor genes with both H3K4me3 modification and RNAPII occupy show higher transcriptional activity than those with only H3K4me3 modification in both HID and MID regions. **c** Four categories of intersection relationships between HID-related and AID-related segments. **d** Microscopic images of H3K9me2- and H3K4me3-immunostaining speckles. H3K9me2 (purple), H3K4me3 (green), DNA stain (DAPI, blue), and overlapped H3K9me2- and H3K4me3-immunostaining speckles (orange) in the rice nucleus. Bar = 2 μm. Data are mean ± s.e.m. *N* = 22. **e** Percentage of RNA-seq reads mapped to the indicated CID regions. **f** Boxplot for transcription levels of expressed genes in four categories of CIDs and gap. NS no significant difference. ***p < 0.001 from Kruskal–Wallis test. **g** Expression levels of H3K4me3 BP and anchor genes located in HID and MID (existing H3K9me2 loops) and/or other CIDs (no H3K9me2 loop). H3K9me2 loops (existed in) have no effect on the expression of H3K4me3-marked BP genes and anchor genes. NS no significant difference. ***p < 0.001 from Kruskal–Wallis test. **h** Percentages of anchor genes and BP genes in the indicated CID modules. **i** The model for chromosomal folding based on H3K9me2, H3K4me3, and RNAPII ChIA-PET data. Zoomed-in region highlights that MID is a transition region between active and inactive domains. **j** Representative chromatin loops from different categories of CIDs and their effects on gene transcription. More red wavy lines indicate higher expression. Boxplots in **d**, **f**, **g** show the median, and third and first quartiles. Source data of **a**, **d**, **f**, **g** are provided as a source data file

We aimed to comprehensively characterize the rice genome architecture with different chromatin interacting domains (CIDs), including HID, AID, mixed interacting domains (MID), TID, and gap regions (Fig. 4a, b). The HID (114 Mb, 32% of the genome), AID (91 Mb, 25%), and TID (73 Mb, 20%) regions were referred to as H3K9me2-, H3K4me3-, or RNAPII-associated CIDs, respectively. A global view of CIDs revealed that the rice genome was spatially partitioned into distinct modules, which were arranged in intervals in rice chromosomes with HIDs enriched in pericentromeric regions (Supplementary Fig. 9). We further identified 385 HID-related and 2231 AID-related segments from our ChIA-PET data. These segments were consistent with microscopic analysis indicating ~316 H3K9me2-immunostaining speckles versus 1096 H3K4me3-immunostaining speckles (Fig. 4c, d), with the assumption that one-immunostaining speckle represents one CID. A possible reason for the smaller number of H3K4me3-immunostaining speckles than that of AID-related segments could be due to the capture power for low fluorescence signals. Strikingly, the majority (77%) of AID-related segments were isolated from HID-related segments (Fig. 4c). The overlapping regions accounted for 5% (19 Mb) of the rice genome and were named as H3K9me2 and H3K4me3 MIDs (Fig. 4a), consistent with the small proportion of H3K9me2 and H3K4me3 overlapping immunostaining speckles (orange) in the rice nucleus (Fig. 4d). Thus, the modules based on three types of ChIA-PET interactomes annotate the majority (82%) of the rice genome and are separated into relatively independent spatial interacting units.

We investigated genomic properties, epigenomic marks, and transcription activity of different CID modules. The results in Supplementary Fig. 10a–f showed that, AID regions displayed the lowest proportion of *Gypsy* and *Copia* retrotransposons and the highest proportion of *Harbinger* and *Mariner* DNA transposons, as well as the highest density of expressed genes. By contrast, the opposite genomic properties were observed in HID regions compared with those in other regions. Consistently, AID regions comprised the lowest DNA methylation level and inactive mark H3K9me2 density and the highest densities of active marks H3K4me3 and H3K27ac and transcribed mark H3K4me1. HID regions, on the other hand, presented the opposite epigenomic properties (Supplementary Fig. 10g–l). Furthermore, the majority of transcription intensity (85%) and expressed regions (79%) were located in the H3K4me3- and/or RNAPII-related modules (Fig. 4a, e). The transcriptional activity was the highest in the AIDs and MIDs, and higher in TID and the lowest in HID and gap regions (Fig. 4f). Consequently, we defined AID and MID as active modules, TID as moderate transcribed modules, and HID and gap regions as inactive or weak-transcribed modules. These results suggested that different module categories represent distinct genomic and epigenomic properties for genome transcription.

We also investigated the effects of chromatin loops on the transcriptional activity of anchor genes and BP genes. Unexpectedly, we found similar expression levels of H3K4me3 BP genes in all module categories (Fig. 4g), indicating that both H3K9me2 loops and H3K4me3 loops do not affect the transcriptional activity of BP genes. The observations that equal transcription activities of H3K4me3 anchor genes (Fig. 4g) and RNAPII anchor genes (Supplementary Fig. 10m) occur both within or without H3K9me2 loops suggested that the formation of heterochromatic looping had no significant effect on the transcriptional regulation of H3K4me3 and RNAPII anchor genes. Furthermore, we confirmed that the transcriptional levels of expressed genes in different modules were correlated with the percentages of anchor genes (Fig. 4f, h). These results suggested that the spatially separated active and inactive compartments (even those with partial overlap in the linear genome) are independent transcriptional units.

Furthermore, we investigated the relationship between TADs and CIDs. Approximately 25% of the rice genome was covered by identified TADs^28^, while the CIDs covered ~82% of the genome (Fig. 4a; Supplementary Fig. 11a). TADs had a median size of 45 kb, which is larger than that of AIDs (28 kb), TIDs (28 kb), and MIDs (25 kb), but smaller than HIDs (63 kb) (Supplementary Fig. 11b). We overlapped the identified TADs with different CIDs. Compared with the proportions of CID coverage in the rice genome, MID (or TID) regions had the similar proportions of coverage in TADs, while AID regions were enriched in TADs and HID regions were decreased in TADs (Supplementary Fig. 11c). Although some TADs coincided well with different types of CIDs (Supplementary Fig. 11a, purple box), most of the TADs covered or overlapped multi-CIDs with distinct genomic properties (Supplementary Fig. 11d, e). Together, these results indicate that TAD is a larger spatial chromatin interaction domain that was composed of various CIDs, while different types of CIDs serve as more accurate sub-TAD domains to annotate the spatial genome architecture.

Collectively, we presented a comprehensive rice 3D model for transcriptional regulation, termed the compartment model (Fig. 4i, j). The rice genome is a hierarchical architecture organized at multiple scales, including chromatin loops, CIDs, compartments, and chromosome territories. The 3D architecture was divided into five modules with distinct genomic and epigenomic properties at the CID scale as follows: AID (active module), HID and gap regions (inactive/weak transcriptional module), MID (active module, transition module between AID- and HID-related segments), and TID (medium transcriptional module). Taken together, these modules are arranged in segments in rice genome (Supplementary Fig. 9) and are organized into independent transcription domains with distinct biological functions.

### Genetic variants alter chromatin topology and transcription

**Fig. 5:**
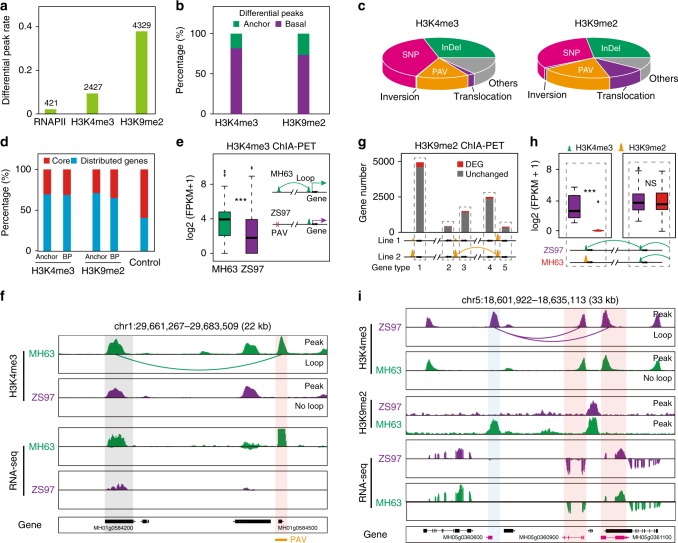
Effects of genetic variations on chromatin topology and transcription. **a** Rates and numbers of H3K4me3 and H3K9me2binding sites and RNAPII occupancy in the two rice varieties MH63 and ZS97. **b** Percentages of H3K4me3 and H3K9me2 differential peaks involved and not involved in chromatin interactions. **c** Pie chart for the proportion of different genetic variation types overlapped with H3K4me3 (upper) and H3K9me2 (bottom) differential peaks involved in interactions. Most (~80%) differential peaks overlapped with single-nucleotide polymorphisms (SNPs), insertion/deletions (InDels), or presence/absence variations (PAVs). **d** Distributions of H3K4me3 and H3K9me2 differential peak-associated core and distributed genes. Percentages of core and distributed genes in 453 rice accessions serve as control. **e** Transcription level of loop-connected and not connected genes in MH63 and ZS97 caused by PAV (present in MH63 and absent in ZS97). ***p *<* 0.001 from Wilcoxon test. **f** Representative screenshot shows a H3K4me3 loop present in MH63 but absent in ZS97 caused by a PAV. The loop-connected gene MH01g0584200 in MH63 is more active than that in ZS97. **g** Expression change status of genes around (±10 kb) H3K9me2 differential peaks. From left to right, the bars represent (1) genes surrounding H3K9me2 differential peaks without interactions, (2) H3K9me2 differential peaks associated loop-outer genes, (3) H3K9me2 differential peaks associated loop-inner genes, (4) H3K9me2 differential peaks associated loop-connected loop-inner genes, (5) H3K9me2 differential peaks associated loop-connected loop-outer genes in turn. The red parts refer to the differentially expressed genes (DEGs), which occupy only a very small proportion (<10%). **h** Effect of H3K4me3 peaks and loops disrupted by H3K9me2 modification on transcription of genes on the indicated locus. Schematic below is a brief model. NS no significant difference. ***p < 0.001 from Wilcoxon test. **i** An example shows one H3K4me3 anchor was replaced by an H3K9me2 peak, causing the loop changes associated with the H3K4me3-marked region. However, there was no influence on the expression of other looped genes. Boxplots in **e**, **h** show the median, and third and first quartiles. Source data of **f**, **i** are provided as a source data file

Since genetic variations can influence inheritance characteristics in the rice genome, we evaluated the effects of genetic variations on histone marks enriched regions between two rice varieties (MH63 and ZS97). The inactive mark H3K9me2 showed a higher rate of differential peak than did the active mark H3K4me3 (Fig. 5a). Interestingly, most of these differential peaks were basal peaks, with only a small proportion of the differential peaks serving as interaction anchors (Fig. 5b). The main reason for these differential epigenetic mark peaks may be associated with single-nucleotide polymorphisms and insertion/deletions, followed by presence/absence variations (PAVs), while inversions and translocations had smaller effects on these changes (Fig. 5c). Most genes related to the differential peaks were distributed genes (Fig. 5d), in consistent with the regions contained the distributed genes varied largely across rice varieties. These observations suggested that genetic variation regions tend to be silent and not involved in chromatin interactions.

To investigate whether genetic variations could alter chromatin topology and transcription, we identified 86 H3K4me3-associated loops present in MH63 but absent in ZS97, which was associated with by PAVs. In MH63, the expression level of genes aggregated through these loops was significantly higher than that in ZS97 (Fig. 5e). For example, the *MH01g0584500* gene is present in MH63 but absent in ZS97, and there was a chromatin interaction between *MH01g0584500* gene and *MH01g0584200* gene in MH63. The expression level of the interacting gene *MH01g0584200* was higher in MH63 (Fig. 5f). Together, these analyses confirmed that functional genetic variations were associated with H3K4me3-associated chromatin topology and gene expression.

Furthermore, we explored the effects of the differential H3K9me2 on chromatin topology and transcription. Regardless of whether the differential H3K9me2 peaks formed loops, the majority of homologous genes near these differential peaks were silenced or stably transcribed in MH63 and ZS97 (Fig. 5g). We also identified 109 H3K4me3-associated loops that were absent in MH63 but present in ZS97 due to the conversion of H3K4me3 to H3K9me2 on a specific anchor site. Unexpectedly, although the gene expression was largely inhibited in the conversion locus in MH63, there was no significant change in the expression of the genes in the interaction locus between MH63 and ZS97 (Fig. 5h, i). One possible explanation for this finding is that the majority of genes (93%) in the interaction locus participated in multiple chromatin interactions at the same time. These results indicated that functional genetic variations could alter H3K9me2-mediated chromatin topology but have little effect on the expression of the interacted genes.

### eQTLs spatially associate with e-traits via chromatin loops

**Fig. 6:**
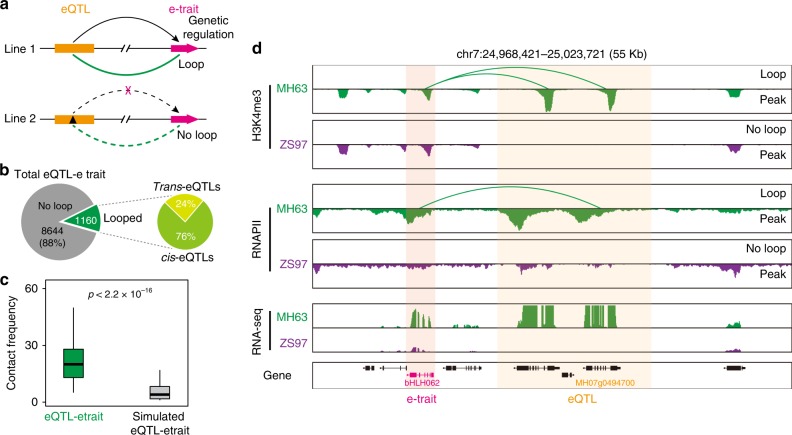
Spatial correlation for the genetic regulation of eQTLs to e-traits. **a** Schematic of expression quantitative trait locus (eQTLs) and expression traits (e-traits) linkages established by ChIA-PET loops. **b** Percentage and numbers of ChIA-PET loop-supported genetic regulation of eQTLs to e-traits (left) and the percentage of *trans*-eQTLs and *cis*-eQTLs (right). **c** Contact frequency of ChIA-PET loop-supported and simulated eQTL-etrait pairs. The distance distribution of simulated eQTL-etrait pairs is similar to that of real eQTL-etrait pairs. The *p* value was calculated by Wilcoxon test. Boxplots show the median, and third and first quartiles. **d** A representative example of ChIA-PET loop-supported eQTLs and e-trait genetic regulation. In MH63, a gene bHLH062 is regulated by an eQTL segment, and they are contacted by H3K4me3- and RNAPII-mediated interactions, while in ZS97, this correlation does not exist. The left highlighted bar shows that this gene exhibits different transcription patterns in rice (MH63 vs ZS97). Source data of **c** are provided as a source data file

Previous genetic analyses revealed that many eQTLs are associated with the expression variation of genes (e-traits) in rice^35,36^. To investigate the potential 3D spatial basis for the genetic regulation of eQTLs on e-traits (Fig. 6a), we collected 9824 eQTLs and e-traits from 210 recombinant inbred lines (RILs) derived from a cross between ZS97 and MH63^36^ (Fig. 6b). From this sample, we detected 1160 (12%) H3K4me3-, RNAPII-, and/or H3K9me2-associated chromatin interactions between eQTLs and e-traits, which is made up of 882 (76%) *cis*-eQTLs and 278 (24%) *trans*-eQTLs (Fig. 6b). Further analysis showed that the contact frequency of these genetic regulations with actual ChIA-PET loops is higher than simulated eQTL-etrait pairs (Fig. 6c), suggesting that spatial connections exist between eQTLs and e-traits. For an example of a spatially connected *trans*-eQTL locus affected by PAV, the generation of spatial connections from an eQTL resulted in higher expression of an e-trait (*bHLH062* gene) (Fig. 6d). Together, these results indicated that spatial physical proximity exists between eQTLs and their associated e-traits, highlighting the genotype effects on spatial interaction and gene expression.

## Discussion

In this study, we constructed the high-resolution 3D architecture of rice genomes using antibodies against RNAPII, active mark H3K4me3, and inactive mark H3K9me2. Approximately 82% of the rice genome was annotated and separated into relatively independent spatial interactive modules, while the spatial independence between active and inactive modules was further validated with an immunofluorescence assay. Notably, 35% of H3K9me2 peaks participated in the inactive modules. In view of the stability of H3K9me2 modification across tissues, the inactive modules may act as scaffolds of the 3D architecture and separate the active modules into different spaces. In the active module, anchor genes involved fundamental biological processes and were organized into different clusters. The distinct transcriptional potential for different module categories mainly depended on the density and relative ratio of basal and anchor genes. Based on these findings, we provide the high-resolution 3D genome organization principles within active and inactive segments, which are separately assigned to different modules or subcompartments with spaced arrangements in the rice high-order genome architecture^28,29^. In addition, it has been recently reported that the dynamics of A/B compartments and TAD domains is associated with the gene expression changes in different tissues^37^. We speculate that active- and inactive-interacting modules identified in this study might also be dynamic between tissues. Previous study reported that the Rabl conformation of rice chromosomes only exist in xylem cells according to FISH experiments^38^, and was hard to observe in Hi-C contact maps in seedlings^28–30,37^. This type of chromosome conformation cannot be detected in our ChIA-PET data. This might be caused by the fact that the 3D genome architecture captured by Hi-C and ChIA-PET method only reflects ensemble structure from different cell types (mesophyll, meristematic, epidermal, vasculature, and guard cells) in rice seedlings.

In contrast with both RNAPII-tethered promoter–enhancer and promoter–promoter interactions in mammals^8,18,19^, we detected only extensive PPIs organized by RNAPII (and H3K4me3) in rice, suggesting that the function of RNAPII varies in higher-order chromatin structure between rice and mammals. One possibility is that the function of mammalian RNAPII to tether promoter–enhancer interactions may be replaced by other factors (such as specific histone modifications or transcription factors) in rice. Another possibility is that there are few typical promoter-enhancer interaction in rice. Consistent with previous studies in mammals^8^, the genetic evidence indicated that promoter fragments in H3K4me3 and/or RNAPII looping enhanced gene transcription. In addition, a higher interaction dimension of the promoters resulted in a higher level of gene expression. Thus, anchor promoters of H3K4me3 and RNAPII may be enhancer-like and play an important role in gene transcription regulation through constructing the rice 3D architecture.

## Methods

### Plant materials and tissue collection

Two rice varieties, ZS97 and MH63 (the parents of one of the most widely cultivated rice hybrids in China, Shanyou 63) were used in this study. The germinating seeds were collected by soaking dry seeds in water for 72 h at 37 °C. The germinated seeds were grown in a phytotron with the day/night cycle set at 14 h/10 h and a temperature of 32 °C/28 °C. Young leaves of 2-week-old seedlings were collected and immediately cross-linked.

### Long-read ChIA-PET library construction and sequencing

Rice long-read ChIA-PET libraries were constructed with a modified ChIA-PET method^6^. Briefly, rice tissues were dual cross-linked with 1% formaldehyde (SIGMA, F8775) for 10 min and 1.5 mM ethylene glycol bis (succinimidylsuccinate) (Thermo Fisher Scientific, 21565) for 30 min at room temperature. The tissues were transferred to 0.2 M glycine for stopping the cross-linking reaction and rinsed three times with ddH2O. Frozen tissues were kept at −80 °C until use. For each experiment, about 4 g of sample was ground in liquid nitrogen into fine powder and resuspended in 100 ml of EB1 buffer (0.4 M sucrose, 10 mM Tris–HCl, 5 mM b-mercaptoethanol, and 1 mM PMSF). The mixed solution was filtered through Miracloth, and the filtrate was centrifuged at 1800 × *g* for 10 min at 4 °C. The pellet was washed three times in 5 ml of EB2 buffer (0.25 M sucrose, 10 mM Tris–HCl, 5 mM b-mercaptoethanol, 10 mM MgCl2, 1% Triton X-100, and 1 mM PMSF) and centrifuged at 2000 × *g* for 10 min at 4 °C. Subsequently, the pellet was resuspended in 2 ml of EB3 buffer (1.7 M sucrose, 10 mM Tris–HCl, 5 mM b-mercaptoethanol, 2 mM MgCl_2_, 0.15% Triton X-100, and 1 mM PMSF) and centrifuged at 2000 × *g* for 1 h at 4 °C. The final pellet was resolved in 1 ml of NLB buffer (10 mM Tris–HCl, 20 mM EDTA, 400 mM NaCl, 1% Triton X-100, and 2 mM PMSF). Sonicate chromatin solution into 1–3 kb (High level, 30 cycles, 30 s ON, and 50 s OFF) using a Bioruptor (Diagenode). Centrifuge the chromatin fragments at 2000 × *g* for 10 min at 4 °C and transfer the supernatant to a new tube for immunoprecipitation. To coat antibodies to magnetic beads, take 60–100 μg antibody (RNAPII, BioLegend, 920102; H3K9me2, Abcam, ab1220 and H3K4me3, ABclonal, A2357) and mix with 800 μl suspended protein G (~1:100 dilution) magnetic beads at 4 °C for 8 h with rotation. Mix sheared chromatin solution to antibody-loaded beads in a new tube at 4 °C overnight with gentle rotation. The supernatant was discarded and the beads were sequentially washed with 5 mL 0.1% SDS FA cell lysis buffer (0.05 M HEPES-KOH, 0.15 M NaCl, 0.001 M EDTA, 1% Triton X-100, 0.1% sodium deoxycholate, and 0.1% SDS) for three times, 5 mL High salt ChIP buffer (0.05 M HEPES-KOH, 0.35 M NaCl, 0.001 M EDTA, 1% Triton X-100, 0.1% sodium deoxycholate, and 0.1% SDS) twice, 5 mL ChIP wash buffer (0.01 M Tris–HCl, 0.25 M LiCl, 0.001 M EDTA, 2.5% NP-40, and 0.5% sodium deoxycholate) once and 5 mL 1 × TE buffer (pH 8.0, Ambion, cat. no. AM9849) twice. ChIP DNA was used for end-repair and A-tailing using T4 DNA polymerase (Promega, cat. no. M421F) and Klenow enzyme (NEB, cat. no. M0212L). Proximity ligation of ChIP DNA was performed using biotinylated bridge-linker (forward strand: 5′-[5Phos]CGCGATATC/iBIOdT/TATCTGACT-3′, reverse strand: 5′-[5Phos]GTCAGATAAGATATCGCGT-3′). Protein-DNA complex was reverse cross-linked and the ChIA-PET libraries were prepared by using Tn5 transposase (VAHTS; cat. no. TD501). The libraries were sequenced (2 × 150 bp) using Illumina Hiseq X-Ten (Beijing AnnoRoad Company).

### Immunofluorescence assay

Rice nuclei isolated from formaldehyde-fixed rice leaves were spread on microscope slides for specimen preparation. Rabbit anti-H3K4me3 (ABclonal, A2357) and mouse anti-H3K9me2 antibodies (Abcam, ab1220), which were used at a 1:200 dilution, were detected with donkey anti-rabbit IgG-Alexa 488 (1:100 dilution, ABclonal, AS035) and goat anti-mouse IgG-TRITC antibodies (1:100 dilution, ABclonal, AS026), respectively. The specimens were counterstained with vectashield antifade mounting medium (Vector, H-1220) and observed under a Nikon N-SIM microscope with 3D SIM mode to scan all the immunofluorescence at different *z*-axis in the whole nuclei. Spot function of Imaris X64 software (www.bitplane.com) was applied to count the foci with an estimated diameter of 0.15 μm and a threshold on “quality”, allowing an accurate detection of visible foci without including the background signal. A total of 22 nuclei were analyzed and averaged for foci counts.

### ChIA-PET data processing

ChIA-PET data were processed with updated ChIA-PET Tool software^39^, a JAVA-based package for automatic processing of ChIA-PET sequence data, including linker filtering, read mapping, redundancy removal, protein-binding sites, and chromatin interaction identification. The genomes were from two rice varieties, MH63 and ZS97, which were downloaded from RIGW. In the ChIA-PET Tool pipeline, we chose ChIP-seq peaks extended by ±10% as the given anchors to call clusters. Considering the technical noise, we identified high-confidence clusters by FDR < 0.05 and a given PET count, which was decided by data sequencing depth. The precise statistics of ChIA-PET replicate data sets and combined data sets are summarized in Supplementary Table 1.

### ChIA-PET contact map construction and normalization

Inter-ligation pets (Ipets: genome span of two tags per pet >8 kb) obtained from H3K4me3- and RNAPII-mediated ChIA-PET unique mapping reads were used to generate raw contact maps with bin size settings of 100, 50, and 10 kb. The raw contact matrix was calculated by the bedpe2Matrix program from ChIA-PET2 software^40^ with “--all --matrix-format complete” parameters. For matrix normalization, we processed the raw contact maps by iterative correction methods from HiC-Pro software^41^ with default settings to adjust the count of the contact matrix. ChIA-PET contact maps were visualized by HiCPlotter software^42^.

### A/B compartment delineating

We used an eigenvector program from juicer software^43^ to delineate A/B compartments in ChIA-PET data at 100, 50, and 10-kb resolution. During this study, each chromosome was divided into fixed windows at coarse resolution. The first principal component of the correlation matrix indicated the compartments.

### Pearson correlation coefficient for co-expression analysis

RNA-seq data of 20 rice tissue samples were collected from the GEO dataset. The detailed information, including GEO accessions and tissue types, is summarized in Supplementary Table 2. Raw reads were mapped to the MH63 reference genome using hisat2^34^ with “--dta-cufflinks” parameters. Mapped reads (.sam format) were further converted to bam format and sorted by read position through samtools^44^. FPKM values were calculated by Cufflinks^45^ with default parameters. The mean value of replicates is referred to as the gene expression level.

According to FPKM values from 20 tissues, we analyzed the co-expression correlation of H3K4me3- and RNAPII-mediated interacting gene pairs (Fig. 2h; Supplementary Fig. 3f). The PCC per anchor gene pair was calculated, and the mean PCC of all interacting anchor gene pairs was considered as the actual co-expression correlation coefficient. Then, we randomly simulated gene pairs 1000 times and set them as controls. Random gene pairs A: randomly simulated gene pairs which have the same physical distance with anchor gene pairs. Random gene pairs B: randomly selected histone-marked gene pairs which have the same physical distance with anchor gene pairs.

As for the co-expression study of subnetworks (Fig. 2l), we selected the top 100 complex subnetworks. For each subnetwork, the mean PCC of all anchor gene pairs in this subnetwork was used as the PCC value of this subnetwork. We compared PCC value of these 100 subnetworks with control.

### Promoter–promoter network analysis

Based on the connection of H3K4me3-associated PPIs, we obtained the top 100 complex subnetworks with intrachromosome pet count ≥4 and interchromosome pet count ≥6 and integrated them into a global network by using Cytoscape (version 3.5.1)^46^ as shown in Supplementary Fig. 4. The network was analyzed and visualized by a layout algorithm, Allegro Spring-Electric, supported by the third-party app, AllegroLayout.

### Clustering of H3K4me3-associated intrachromosomal loops

We performed k-means clustering for H3K4me3-associated intrachromosomal interactions by Deeptools^47^. The loop region for clustering was extended by ±10% loop length. According to the transcription factor modification patterns within H3K4me3 loops, we classified all loops into seven groups, as shown in Supplementary Fig. 7.

### Identification of chromosome interaction domains

We first identified multiple chromatin loops that are connected to each other and clustered into candidate domains with continuous chromatin loop coverage. We further calculated the aggregate loop coverage along all chromosomes at base-pair resolution and subtracted these low-coverage regions from the candidate domains. Subsequently, the resulting domain regions were filtered by a genomic span. The domains more than 1 Mb in size (200 kb for AIDs) are excluded and yielded the final TIDs and HIDs. A CID means a genomic region spanned by continuous connected loops mediated by specific protein factor. Four categories of CIDs were defined: H3K9me2-associated HIDs, H3K4me3-associated AIDs, and MIDs covered by both H3K9me2 and H3K4me3, and only RNAPII-associated TIDs.

### Identification of differential peaks

We used a merge function from bedtools software^48^ to combine MH63 peaks (MH63 ChIP-seq data map to MH63) and ZS97 peaks (ZS97 ChIP-seq data map to MH63), which overlap by at least 1 bp. Differential peaks were calculated by the DESeq package^49^ based on the merged binding peaks. The parameters for differential peaks were adjusted *p*-value < 0.05, log2-fold-change >1 or <−1. The numbers of differential peaks are shown in Fig. 5a.

### Reporting Summary

Further information on research design is available in the Nature Research Reporting Summary linked to this article.

## Supplementary information

Supplementary information

Reporting Summary

Source data

## Data Availability

Data supporting the findings of this work are available within the paper and its Supplementary Information files. A reporting summary for this Article is available as a Supplementary Information file. The sequence data are available at NCBI GEO under accession number GSE131202. All accessions of published RNA-seq data used in this study are provided in Supplementary Table 2. The source data underlying Figs. 1e, f, 2d, k, 3a, d, 4a, d, f, g, 5f, i, and 6c, and Supplementary Figs. 3b, 6c, and 7c, d are provided as a Source Data file. All other data generated and analyzed during the current study are available from the corresponding author upon reasonable request.
