# Supplementary material for: Chromatin loops associated with active genes and heterochromatin shape rice genome architecture for transcriptional regulation
Source: Nat Commun. 2019 Aug 13;10:3640. doi: 10.1038/s41467-019-11535-9 (PMC6692402; doi:10.1038/s41467-019-11535-9)
Supplement: Supplementary file 1 — Supplementary information [file 41467_2019_11535_MOESM1_ESM.pdf]

Chromatin loops associated with active genes and heterochromatin shape rice genome architecture for transcriptional regulation

Zhao *et al.*

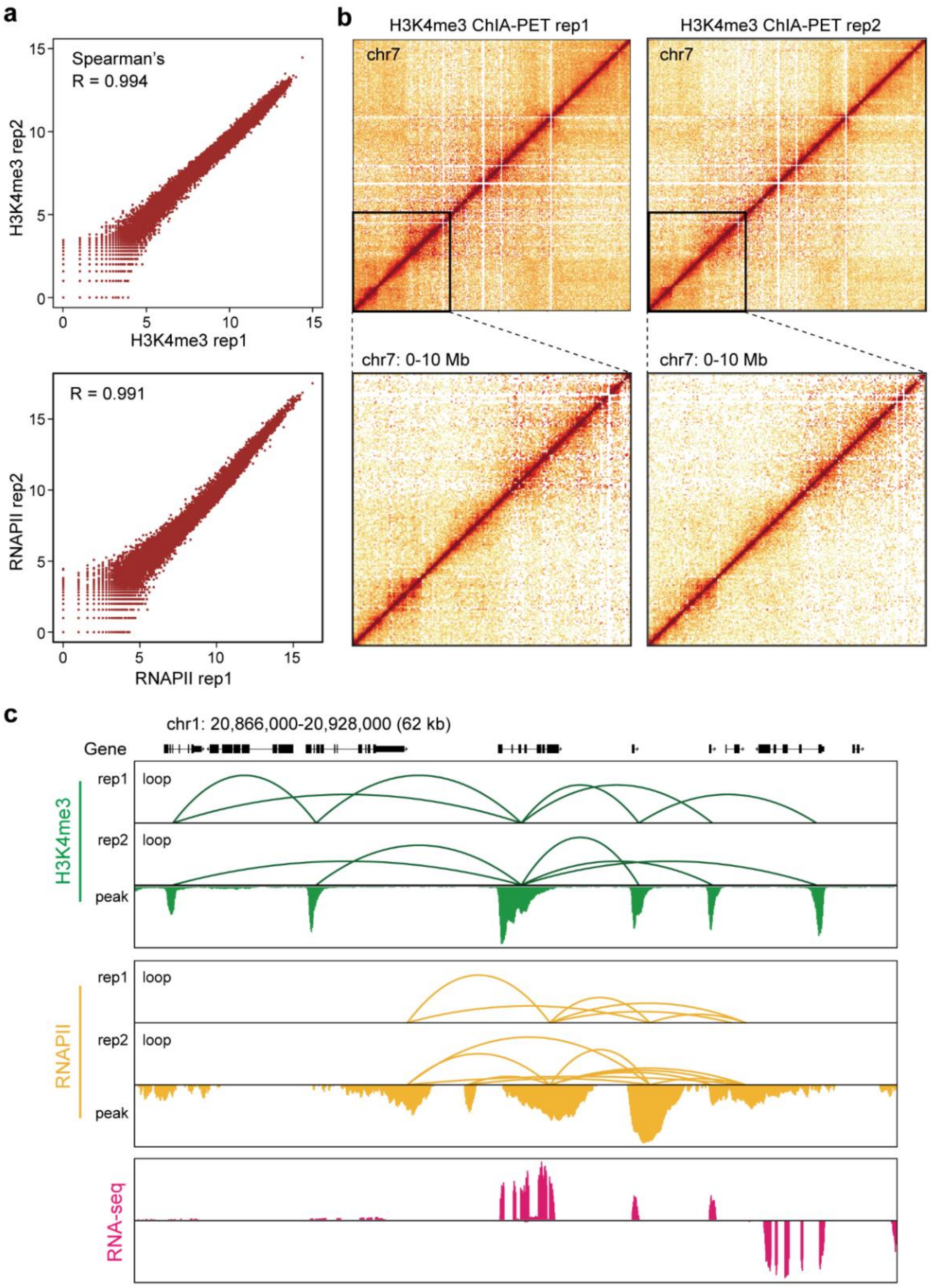

Supplementary Fig. 1. ChIA-PET data reproducibility.

a, Scatter plots showing the reproducibility between two ChIA-PET replicates in MH63. Upper: H3K4me3-associated ChIA-PET data; lower: RNAPII-associated ChIA-PET data. Spearman correlation coefficients are shown. **b,** Comparison of ChIA-PET interaction heatmaps between two H3K4me3 replicates in MH63 represented by chromosome 7. Upper: 100-kb resolution; lower: zoomed-in views at 50-kb resolution. **c,** Genome browser at the indicated region shows the high reproducibility of ChIA-PET data.

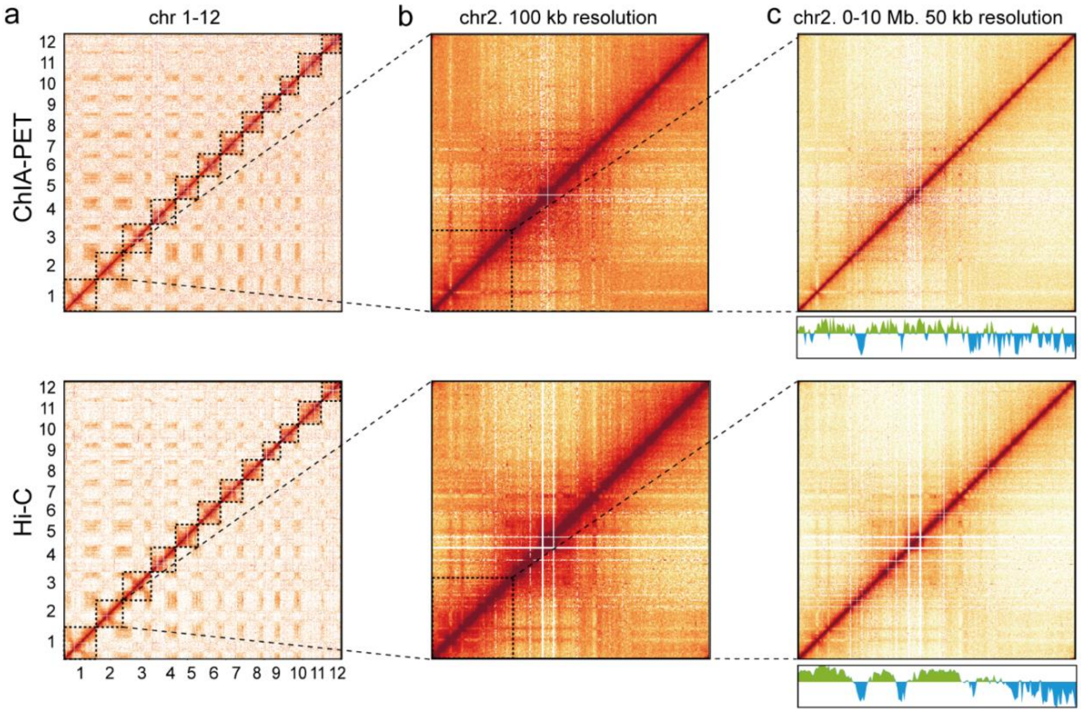

d

Spearman's correlation coefficient between ChIA-PET and Hi-C data

chr.	1	2	3	4	5	6	7	8	9	10	11	12
	0.768	0.709	0.811	0.671	0.538	0.731	0.695	0.627	0.666	0.693	0.664	0.707

Supplementary Fig. 2. Comparison between ChIA-PET and *in situ* Hi-C data in rice.

a, Genome-wide contact heatmaps at 100-kb resolution from RNAPII and H3K4me3 with ChIA-PET data (this study) and Hi-C data (previous study) combined. **b,** ChIA-PET and Hi-C maps at 100-kb resolution represented by chr2 showing similar contact patterns. **c,** Zoomed-in views for 0-10 Mb of chr2 at 50-kb resolution showing similar contact patterns. The principal component (PC) 1 values are also shown at the bottom. **d,** Spearman's correlation coefficient between ChIA-PET and Hi-C interaction heatmap matrices (200 kb × 200 kb) per chromosome.

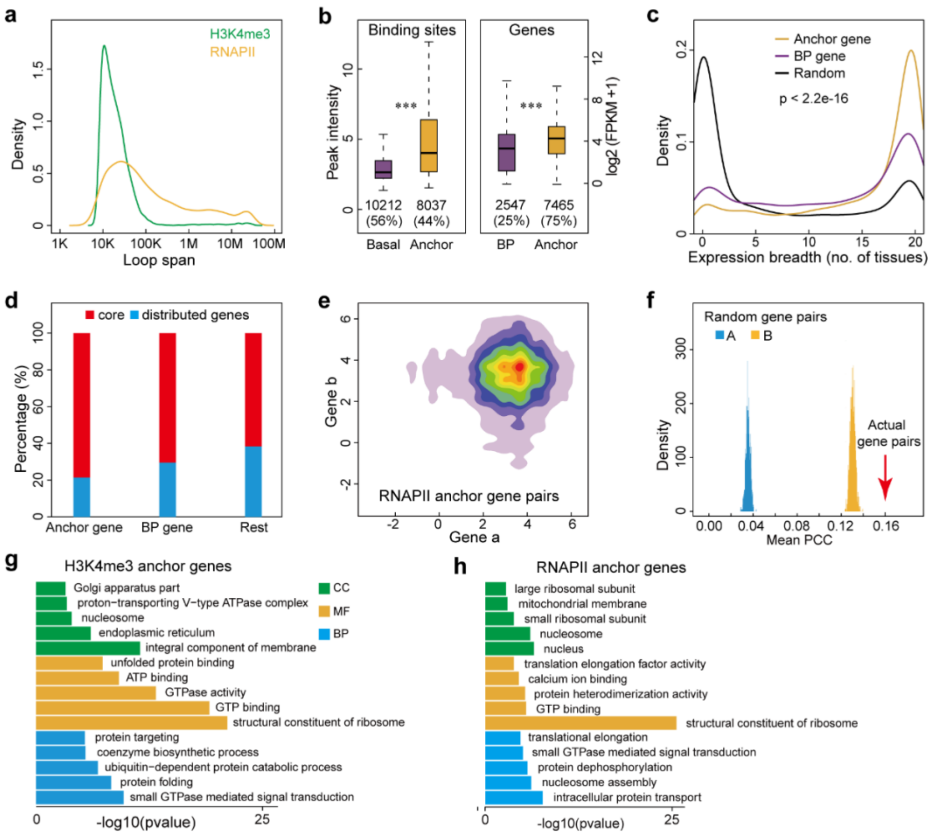

Supplementary Fig. 3. Features of RNAPII binding peaks and chromatin loops in rice.

a, Loop span distribution of H3K4me3- and RNAPII-associated chromatin interactions. **b,** Boxplots show the intensities of RNAPII peaks (left) and expression levels of genes (right) involved (anchor) and not involved in (basal) interactions. Numbers and percentages are shown. *** for p < 0.001 from Wilcoxon test. Boxplots show the median, and third and first quartiles. Source data are provided as a Source Data file. **c,** Expression breadth of RNAPII-associated anchor and BP genes. Random genes served as control. p < 2.2e-16 from Kruskal-Wallis test. **d,** Distribution of core and distributed genes in RNAPII-associated chromatin models. **e,** Contour plot of log-transformed FPKM values for co-transcription of RNAPII anchor gene pairs. **f,** Histogram for co-expression analysis of RNAPII anchor gene pairs. The mean Pearson correlation coefficient (PCC) of anchor gene pairs is much higher than that of both randomly simulated gene pairs (A) which have the same physical distance with anchor gene pairs and randomly selected RNAPII-marked gene pairs which have the same physical distance with anchor gene pairs (B). **g-h,** Top five GO terms of anchor genes at three aspects. The bar length represents -log10 (p-value). CC, Cellular component; MF, Molecular function; BP, Biological process. Source data of Supplementary Fig. 3b are provide as a Source Data file.

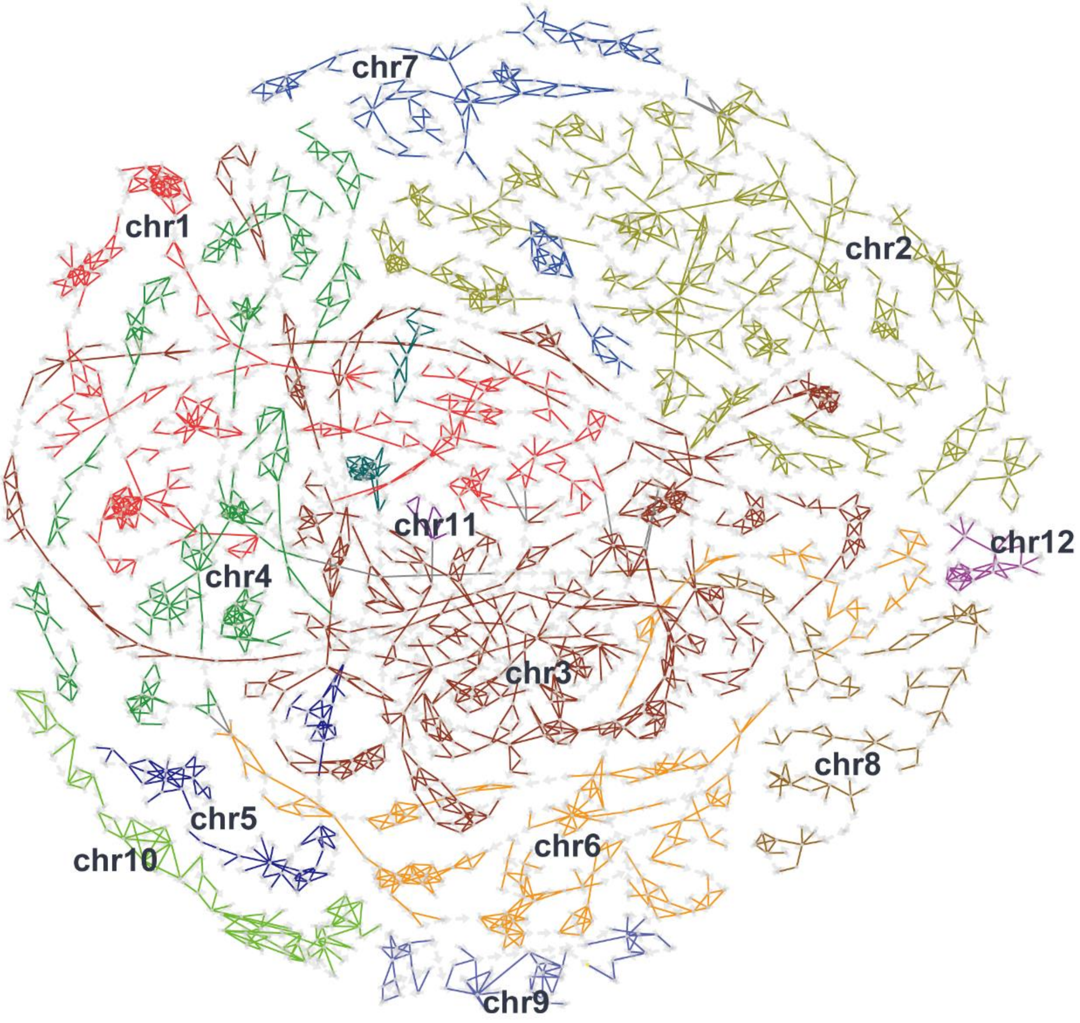

Supplementary Fig. 4. Global view of H3K4me3-mediated genome interacting structure. Network showing overall promoter-promoter interactions identified by H3K4me3 ChIA-PET data, with different chromosomes in different colors. Each chromosome index is marked on its chromatin territory.

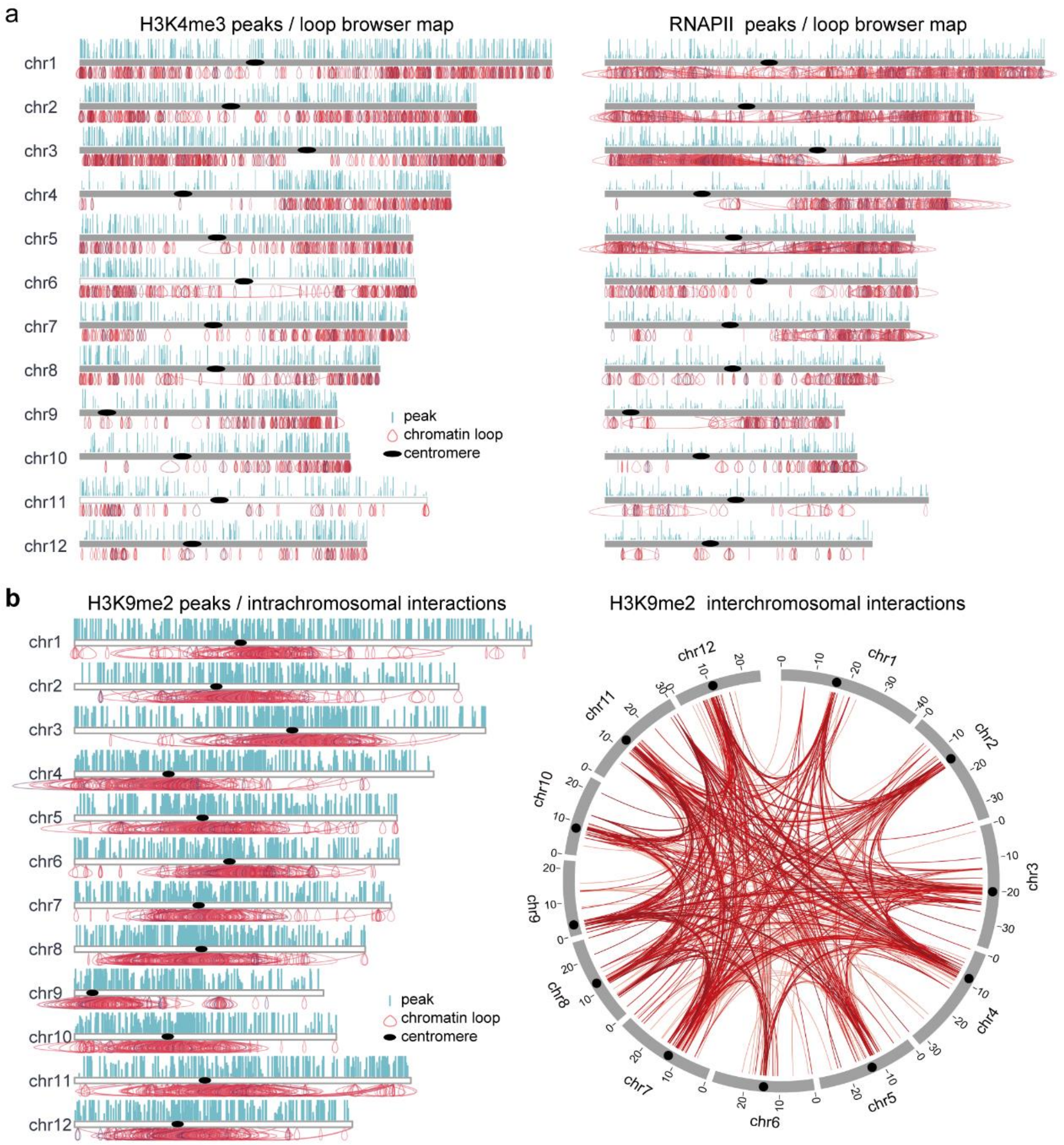

Supplementary Fig. 5. Global patterns of chromatin interactions.

a, Global views of intrachromosomal interactions from H3K4me3 (left) and RNAPII (right) ChIA-PET data. Blue peaks above chromosomes are binding sites, and red curves under chromosomes are interactions. Redder colors indicate stronger interactions. **b,** Global view of intrachromosomal and interchromosomal interactions mediated by H3K9me2 marked regions. The green bars in graph refer to H3K9me2 binding sites. Red curve refers to interactions; redder colors indicate stronger interactions.

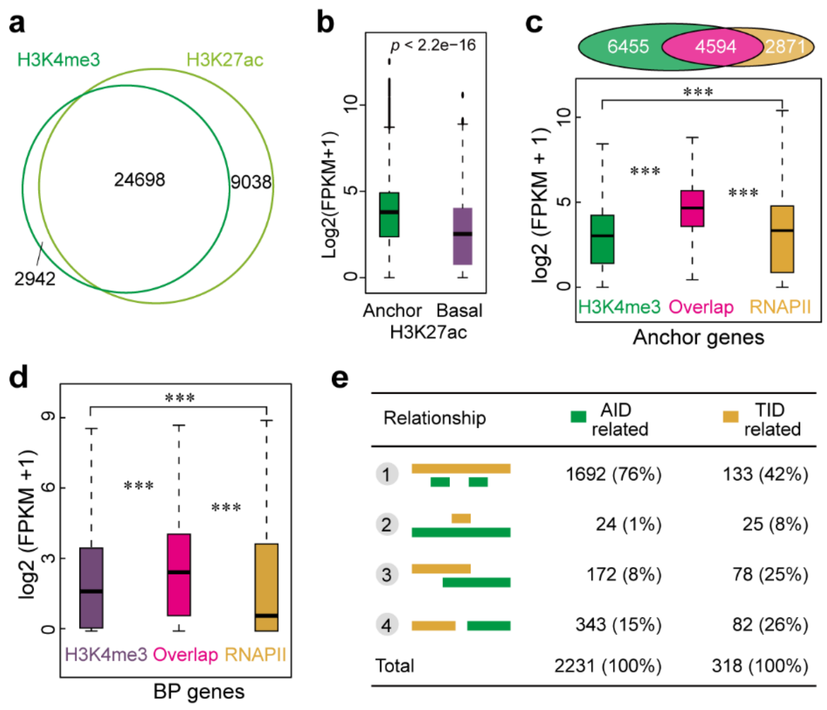

Supplementary Fig. 6. Relationship of active gene associated chromatin interactions.

a, Venn diagram showing the overlap of H3K4me3 and H3K27ac binding sites. **b,** Boxplots for expression levels of H3K27ac-marked anchor and basal genes. H3K27ac-marked anchor and basal genes were H3K4me3-marked anchor and basal genes overlapped with H3K27ac peaks, respectively. **c,** Expression level of anchor genes marked by H3K4me3 only, RNAPII only or both. Venn diagram shows that 62% of RNAPII anchor genes overlap with H3K4me3 anchor genes. Source data are provided as a Source Data file. **d,** Expression level of BP genes marked by H3K4me3 only, RNAPII only or both. Genes labeled with both RNAPII and H3K4me3 have the highest expression intensity. *** for $p < 0.001$ from Wilcoxon test. **e,** Four categories of intersection relationships between AID-related and TID-related segments. AID-related, H3K4me3-related active interacting domains, TID-related, RNAPII-related transcriptional interacting domains. Boxplots in Supplementary Fig. 7a, b show the median, and third and first quartiles. Source data of Supplementary Fig. 6c are provide as a Source Data file.

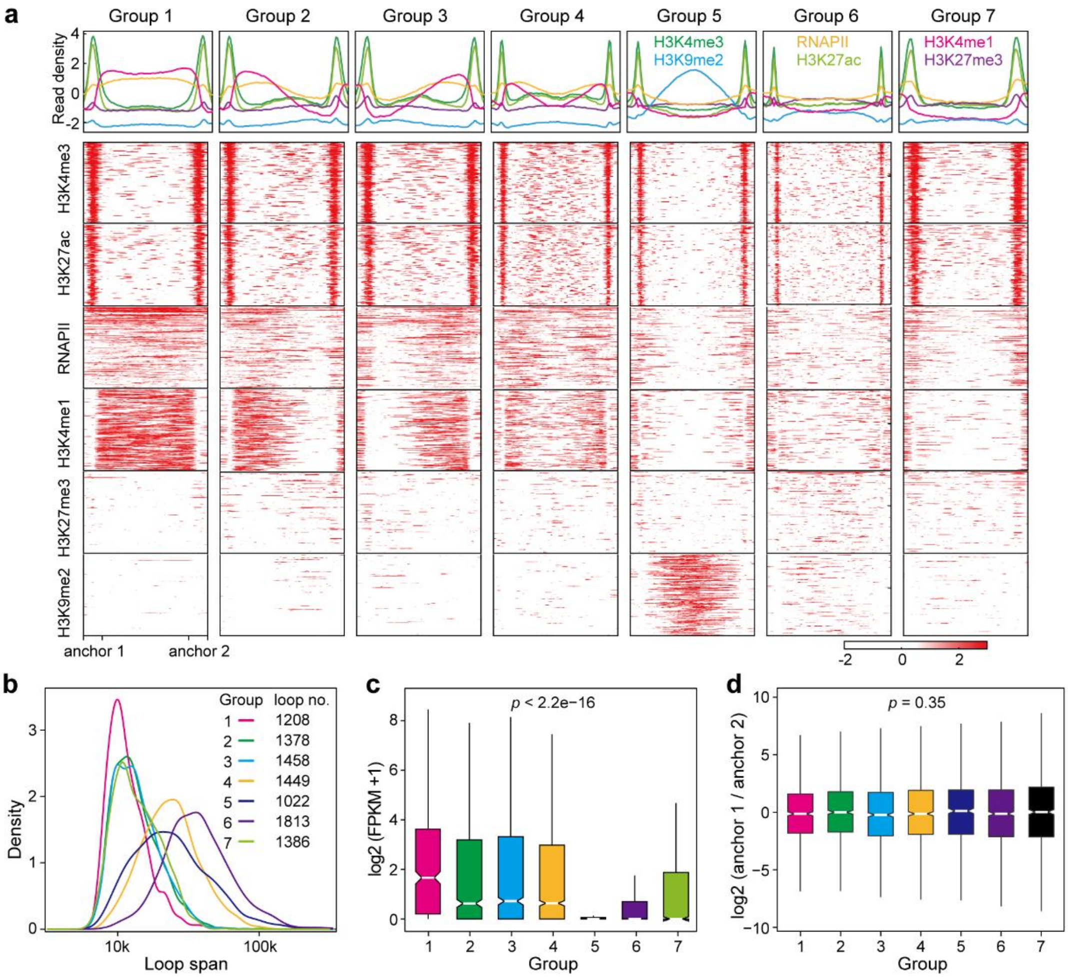

Supplementary Fig. 7. Epigenome features of H3K4me3 loop regions.

a, H3K4me3-associated loop patterns defined by five histone modifications and RNAPII. Heatmaps showing the histones and RNAPII enrichment signals for all loops as well as their extended regions (±0.1 loop length). **b,** Density plot for the loop span distributions of seven groups in **a**. Group 1 presents the lowest span, while Group 6 presents the highest span. **c,** Boxplot showing the normalized expression level of loop genes (genes within loops, exclude anchor genes) of each group. p < 2.2e-16 from Kruskal-Wallis test. **d,** Comparison for relative expression intensity of anchor genes at each end of the loop in seven clusters. p = 0.35 from Kruskal-Wallis test. Boxplots in Supplementary Fig. 6c, d show the median, and third and first quartiles. Source Data of Supplementary Fig. 7c and 7d are provided as a Source Data file.

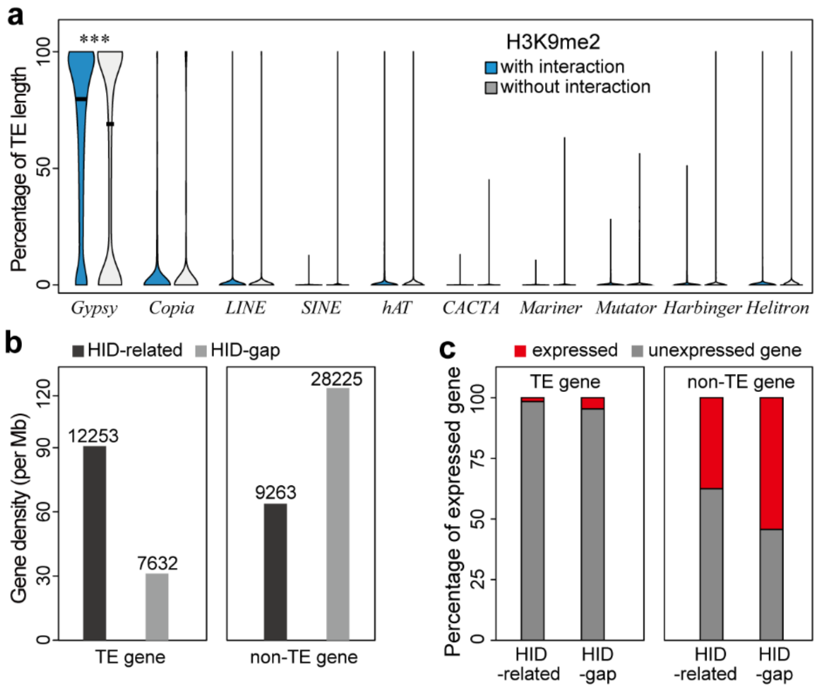

Supplementary Fig. 8. Genomic features of H3K9me2-associated chromatin interactions.

a, TE occupancy (%) of H3K9me2 binding sites involved and not involved in interactions. *** for $p < 0.001$ from Wilcoxon test. **b,** Densities of TE gene (left) and non-TE gene (right) located in HID-related and HID-gap regions, respectively. Gene numbers are also shown. **c,** Percentage of expressed (red) and unexpressed genes (gray) located in the HID-related region and HID-gap region. Left: TE gene; right: non-TE gene.

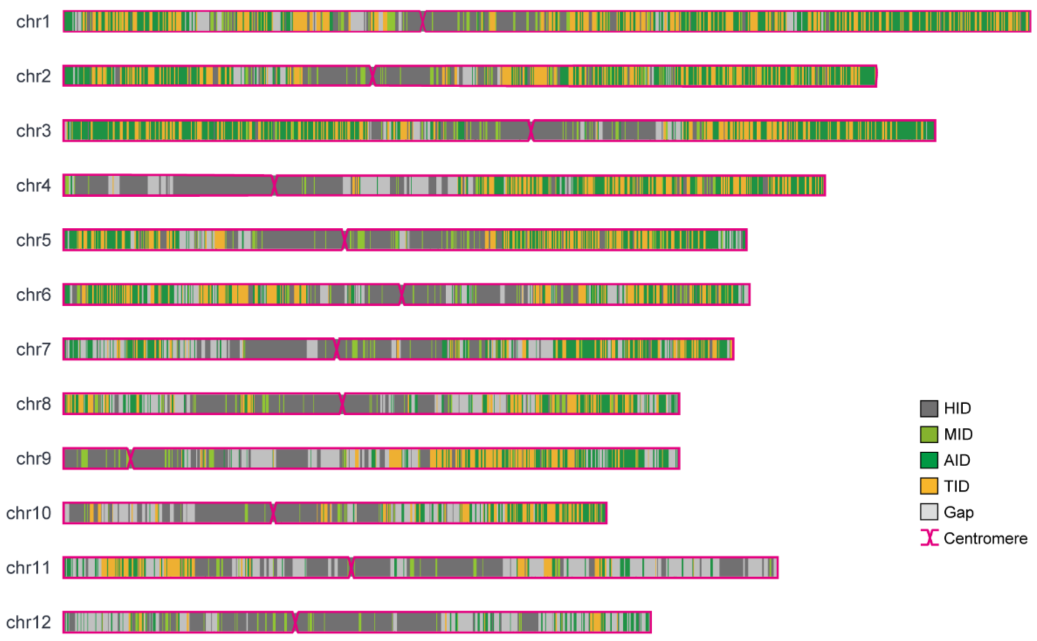

Supplementary Fig. 9. Global view of different CIDs in each rice chromosome.

Combinatorial pattern in MH63 showing interval arrangement by different CIDs in rice chromosomes. Overall, HIDs are enriched in centromeres and pericentromeric regions. H3K9me2-mediated heterochromatic interacting domains (HIDs), H3K4me3-mediated active interacting domains (AIDs), mixed interacting domains mediated by both H3K9me2 and H3K4me3 (MIDs), and only RNAPII-mediated transcriptional interacting domains (TIDs).

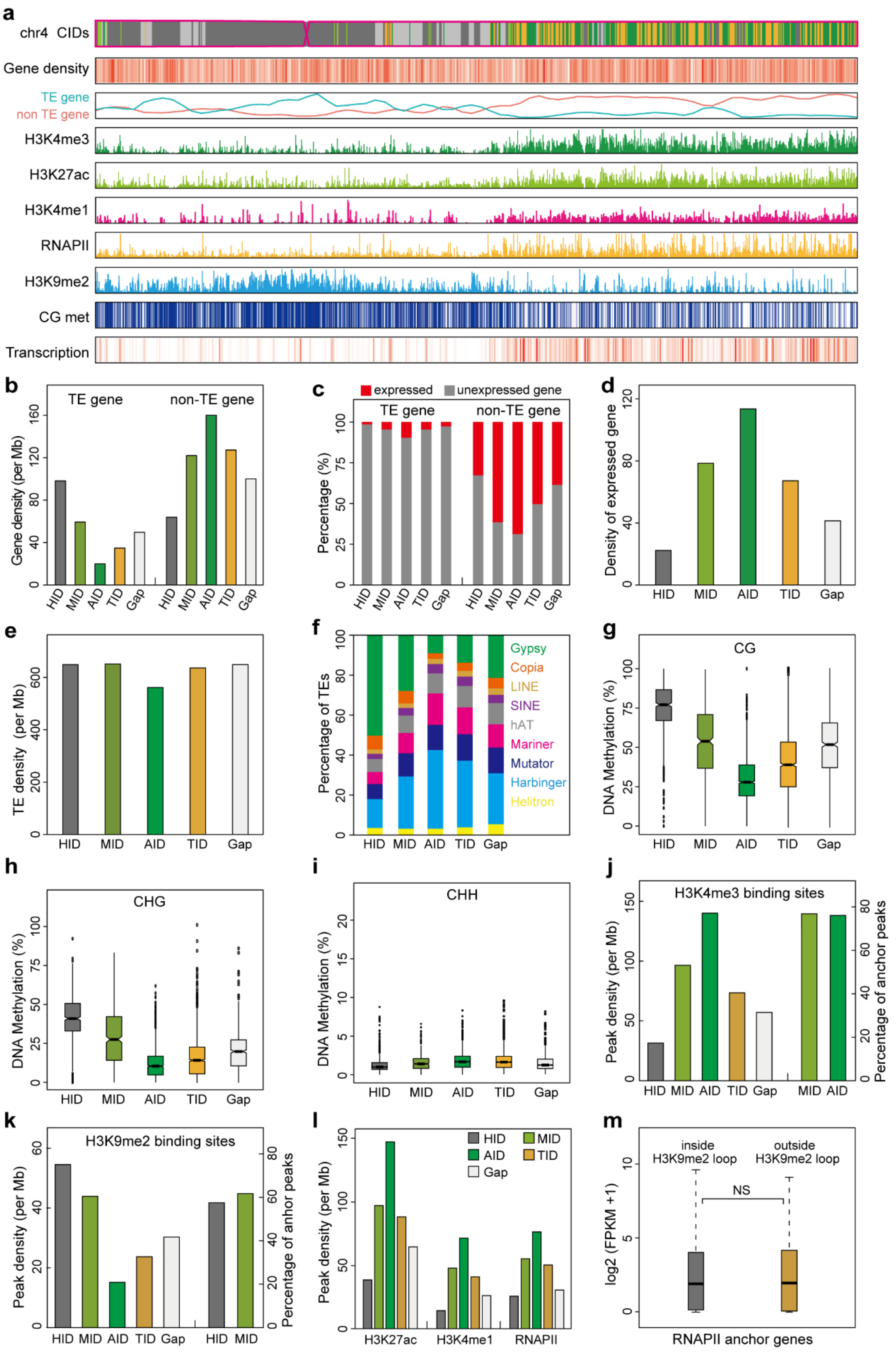

Supplementary Fig. 10. Genomic and epigenetic properties of CIDs.

a, Colocalization of CIDs, genomic composition and representative epigenetic marks and

transcriptional features within chr4. Densities of genes, epigenetic modifications, CG methylation and gene transcription of chr4 are shown in 50-kb bins. **b,** Densities of TE genes and non-TE genes in different CID categories. **c,** Percentages of expressed genes and unexpressed genes in different CID categories. Left: TE gene; right: non-TE gene. **d,** Densities of expressed genes in different CID categories. **e,** Densities of TEs in different CID categories. **f,** Percentages of different TE components in different CID categories. **g-i,** DNA methylation levels in different CID categories. CG methylation in g, CHG methylation in h, CHH methylation in i. AID shows the lowest, while HID shows the highest. **j,** Densities of H3K4me3 binding sites in different CID categories (left) and the percentages of H3K4me3 anchor peaks in MID and AID (right). **k,** Densities of H3K9me2 binding sites in different CID categories (left) and the percentages of H3K9me2 anchor peaks in HID and MID (right). **l,** Densities of H3K27ac, H3K4me1, and RNAPII binding sites in different CID categories. **m,** Effect of H3K9me2 loop on the expression of RNAPII anchor genes showing that the H3K9me2 loop does not affect the transcription of RNAPII anchor genes. Wilcoxon test, NS, no significant difference. Boxplots in Supplementary Fig. 10g-i, m show the median, and third and first quartiles.

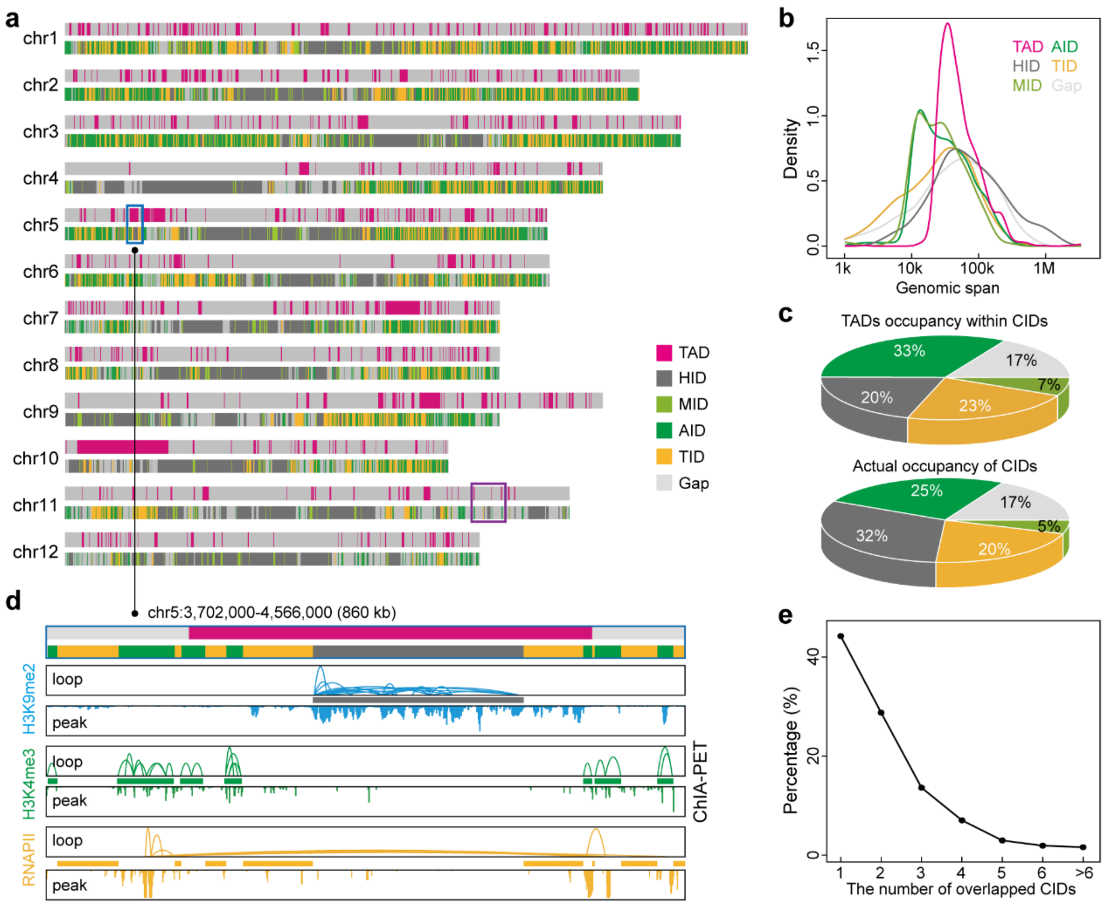

Supplementary Fig. 11. Comparison of CIDs in this study with TADs identified by Hi-C data. **a,** Global distribution of CIDs and TADs in each rice chromosome (MH63). The purple box represented TAD regions that coincide well with CID regions. The blue box represented one TAD covered or overlapped multiple CIDs. **b,** Genomic span distribution of CIDs and TADs. **c,** TADs occupancy within CIDs (upper) and actual occupancy of CIDs (lower). **d,** Zoomed-in view of blue box of a showed an example for a TAD overlapping multiple CIDs. **e,** Percentage of TADs overlapping different number of CIDs.

Supplementary Table 1. Summary of ChIA-PET libraries.

Variety	Factor	Replicate	Total PETs	Uniquely Mapped PETs	Remove redundancy	Self-ligation PETs	Inter-ligation PETs	Other PETs	PET count cutoff	Clustered PET	PET count cutoff	Clustered PET	Intra-chrom cluster	Inter-chrom clusters
MH63	H3K4me3	Combined	138,491,629	57,859,019	44,478,609	17,643,287	23,390,518	3,444,804	2	373,621	4	11,230	9,714	1,516
		Replicate 1	81,072,389	34,085,399	25,545,916	9,297,209	14,517,148	1,731,559	2	65,438				
		Replicate 2	57,419,240	23,773,620	18,932,693	8,346,078	8,873,370	1,713,245	2	30,605				
ZS97	H3K4me3	Combined	147,370,998	34,977,054	25,148,232	12,405,117	10,472,033	2,271,082	2	128,426	4	12,887	12,279	608
		Replicate 1	74,730,098	24,464,399	17,300,923	9,256,273	6,520,205	1,524,445	2	53,757				
		Replicate 2	72,640,900	27,372,086	10,711,137	4,672,079	5,141,673	897,385	2	48,770				
MH63	RNAPII	Combined	160,000,819	57,826,952	29,662,712	15,955,097	10,144,177	3,563,438	2	105,583	4	28,213	17,160	11,053
		Replicate 1	92,291,446	29,020,027	16,653,684	7,137,684	7,226,665	2,289,335	2	77,064				
		Replicate 2	67,709,373	28,806,925	13,009,028	8,817,413	2,917,512	1,274,103	2	41,738				
ZS97	RNAPII	Combined	393,873,765	128,514,139	80,626,657	58,419,946	17,033,578	5,173,133	2	391,451	5	26,570	13,012	13,558
		Replicate 1	237,823,921	70,815,647	46,337,758	33,192,034	9,827,959	3,317,765	2	231,470				
		Replicate 2	156,049,844	57,698,492	34,288,899	25,227,912	7,205,619	1,855,368	2	222,063				
MH63	H3K9me2	Combined	222,944,767	19,976,849	16,486,622	4,418,718	10,979,578	1,088,326	2	49,885	4	11,307	5,627	5,680
		Replicate 1	94,444,032	8,567,004	7,273,687	1,695,022	5,095,515	483,150	2	47,780				
		Replicate 2	128,500,735	11,409,845	9,212,935	2,723,696	5,884,063	605,176	2	49,315				
ZS97	H3K9me2	Combined	394,192,536	35,499,121	26,301,538	6,311,948	17,988,084	2,001,506	2	33,421	4	12,517	6,934	5,583
		Replicate 1	216,918,079	19,336,746	12,816,533	2,787,407	8,850,978	1,178,148	2	38,176				
		Replicate 2	177,274,457	16,162,375	13,485,005	3,524,541	9,137,106	823,358	2	33,807				

The ChIA-PET datasets of MH63 and ZS97 in the above table were mapped to their own reference genome.

Supplementary Table 2. RNA-Seq data for different tissues used in this study.

Tissue number	Tissue	SRA accession	SRR accession	GEO accession	Varieties
1	Young leaf (two-week-old)	This study			*O. sativa* L. ssp. *indica* (cv. MH63)
2	Flag leaf before flower transformation	This study			MH63
3	Panicle (1.5-4.5 cm)	This study			MH63
4	One-week-old root	This study			MH63
5	Four-week-old root	SRX2448847	SRR5134063	GSM2441710	*O. sativa* L. ssp. *japonica* (cv. Chilbo)
		SRX2448848	SRR5134064	GSM2441711	*O. sativa* L. ssp. *japonica* (cv. Chilbo)
6	Calli (embryonic stage)	SRX1815135	SRR3724615	GSM2184192	*O. sativa* L. ssp. *japonica* (cv. TNG67)
		SRX1815136	SRR3724616	GSM2184193	*O. sativa* L. ssp. *indica* (cv. IR64)
7	Endosperm	SRX1546415	SRR3123479	GSM2056926	Hybrid of *indica* (cv. Longtefu) and *japonica* (cv. 02428)
		SRX1546416	SRR3123481	GSM2056926	Hybrid of *indica* (cv. Longtefu) and *japonica* (cv. 02428)
8	Nodes I and II	SRX856969	SRR1777239	GSM1594104	*O. sativa*
		SRX856969	SRR1777240	GSM1594104	
9	Stem	SRX856970	SRR1777241	GSM1594105	*O. sativa*
		SRX856970	SRR1777242	GSM1594105	
10	Flower buds before flowering	SRX507920	SRR1213690	GSM1361891	*O. sativa* L. ssp. *japonica* (cv. Nipponbare)
11	Flowers at the flowering day	SRX507921	SRR1213691	GSM1361892	Nipponbare
12	Milk grains	SRX507926	SRR1213696	GSM1361897	Nipponbare
13	Mature seeds	SRX507927	SRR1213697	GSM1361898	Nipponbare
14	Panicle (0.3-1.5 cm)	SRX332586	SRR1633182	GSM1203190	*O. sativa* L. ssp. *japonica* (cv. DongJin)
		SRX332587	SRR1633187	GSM1203191	DongJin
15	Egg cell	SRX348612	SRR976335	GSM1229035	*O. sativa* L. ssp. *japonica* (cv. Kitaake)
		SRX348613	SRR976336	GSM1229036	
		SRX348614	SRR976337	GSM1229037	
16	Sperm cell	SRX348615	SRR976338	GSM1229038	*O. sativa* L. ssp. *japonica* (cv. Kitaake)
		SRX348616	SRR976339	GSM1229039	
		SRX348617	SRR976340	GSM1229040	

Supplementary Table 2. RNA-Seq data for different tissues used in this study (continued).

Tissue number	Tissue	SRA accession	SRR accession	GEO accession	Varieties
17	Vegetative cell	SRX348618	SRR976341	GSM1229041	*O. sativa* L. ssp. *japonica* (cv. Kitaake)
		SRX348619	SRR976342	GSM1229042	
		SRX348620	SRR976343	GSM1229043	
18	Three-week-old leaf	SRX235856	SRR711322	GSM1081573	Nipponbare
		SRX235857	SRR711323	GSM1081574	
19	Three-week-old calli	SRX103307	SRR358795	GSM823083	Nipponbare
		SRX103308	SRR358797	GSM823084	
20	Lamina joints of flag leaf	SRX348482	SRR976168	GSM1229044	Nipponbare
