# Supplementary material for: Chromatin loops associated with active genes and heterochromatin shape rice genome architecture for transcriptional regulation
Source: Nat Commun. 2019 Aug 13;10:3640. doi: 10.1038/s41467-019-11535-9 (PMC6692402; doi:10.1038/s41467-019-11535-9)
Supplement: Supplementary file 2 — Reporting Summary [file 41467_2019_11535_MOESM2_ESM.pdf]

nature research

Corresponding author(s): Xingwang Li

Reporting Summary

Nature Research wishes to improve the reproducibility of the work that we publish. This form provides structure for consistency and transparency in reporting. For further information on Nature Research policies, see Authors & Referees and the Editorial Policy Checklist.

Statistical parameters

When statistical analyses are reported, confirm that the following items are present in the relevant location (e.g. figure legend, table legend, main text, or Methods section).

n/a	Confirmed	
☐	☒	The exact sample size (n) for each experimental group/condition, given as a discrete number and unit of measurement
☐	☒	An indication of whether measurements were taken from distinct samples or whether the same sample was measured repeatedly
☐	☒	The statistical test(s) used AND whether they are one- or two-sided<br>*Only common tests should be described solely by name; describe more complex techniques in the Methods section.*
☒	☐	A description of all covariates tested
☒	☐	A description of any assumptions or corrections, such as tests of normality and adjustment for multiple comparisons
☐	☒	A full description of the statistics including central tendency (e.g. means) or other basic estimates (e.g. regression coefficient) AND variation (e.g. standard deviation) or associated estimates of uncertainty (e.g. confidence intervals)
☐	☒	For null hypothesis testing, the test statistic (e.g. F, t, r) with confidence intervals, effect sizes, degrees of freedom and P value noted<br>*Give P values as exact values whenever suitable.*
☒	☐	For Bayesian analysis, information on the choice of priors and Markov chain Monte Carlo settings
☒	☐	For hierarchical and complex designs, identification of the appropriate level for tests and full reporting of outcomes
☐	☒	Estimates of effect sizes (e.g. Cohen's d, Pearson's r), indicating how they were calculated
☐	☒	Clearly defined error bars<br>*State explicitly what error bars represent (e.g. SD, SE, CI)*

Our web collection on statistics for biologists may be useful.

Software and code

Policy information about availability of computer code

Data collection	All sequencing libraries were prepared in house and raw reads were generated on Illumina high-throughtput sequencing paltform with manufacture's instruction. The RNA-seq data used for gene expression breadth are published datasets and summarized in Supplementary Table 2.
Data analysis	Software used include: ChIA-PET tool, ChIA-PET2, HiC-Pro, HiCPlotter, Juicer, HISAT, Cufflinks, SAMtools, Cytoscape, deepTools, BEDTools, DESeq as well as R version 3.2.5 and Perl to run many of the mentioned programs. Detailed parameters of each of the programs are mentioned in relevant sections in Methods.

For manuscripts utilizing custom algorithms or software that are central to the research but not yet described in published literature, software must be made available to editors/reviewers upon request. We strongly encourage code deposition in a community repository (e.g. GitHub). See the Nature Research guidelines for submitting code & software for further information.

Data

Policy information about availability of data

All manuscripts must include a data availability statement. This statement should provide the following information, where applicable:

- Accession codes, unique identifiers, or web links for publicly available datasets
- A list of figures that have associated raw data
- A description of any restrictions on data availability

All of raw data has been uploaded to NCBI GEO under accession code GSE131202 and are publicly available now via the link (https://www.ncbi.nlm.nih.gov/geo/query/acc.cgi?acc=GSE131202).

Field-specific reporting

Please select the best fit for your research. If you are not sure, read the appropriate sections before making your selection.

[x] Life sciences [ ] Behavioural & social sciences [ ] Ecological, evolutionary & environmental sciences

For a reference copy of the document with all sections, see nature.com/authors/policies/ReportingSummary-flat.pdf

Life sciences study design

All studies must disclose on these points even when the disclosure is negative.

Sample size	No statistical methods were used to predetermine sample size.
Data exclusions	No exclusion of data was made.
Replication	All experimental data was reliably reproduced in multiple independent experiments as indicated in the figure legends.
Randomization	Randomization was not used.
Blinding	No blinding was used.

Reporting for specific materials, systems and methods

Materials & experimental systems

n/a	Involved in the study	
x		Unique biological materials
	x	Antibodies
x		Eukaryotic cell lines
x		Palaeontology
x		Animals and other organisms
x		Human research participants

Methods

n/a	Involved in the study	
x		ChIP-seq
x		Flow cytometry
x		MRI-based neuroimaging

Antibodies

Antibodies used	Antibodies used for ChIA-PET and Immunofluorescence assay: H3K4me3 polyclonal antibody (ABclonal, A2357), H3K9me2 monoclonal antibody (Abcam, ab1220), RNAPII monoclonal antibody (BioLegend, 920102), donkey anti-rabbit IgG-Alexa 488 (ABclonal, AS035) and goat anti-mouse IgG-TRITC antibodies (ABclonal, AS026).
Validation	The antibodies have been validated by the company and our lab using western blot, dot plot, Immunofluorescence and ChIP-seq experiments. These commercial antibodies are well used and reported in lots of previous publications of our and other labs. Please see information of antibodies below:<br>H3K4me3 antibody: https://www.abclonal.com.cn/catalog/A2357;<br>H3K9me2 antibody: https://www.abcam.cn/histone-h3-di-methyl-k9-antibody-mabcam-1220-chip-grade-ab1220.html;<br>RNAPII antibody: https://www.biolegend.com/.<br>donkey anti-rabbit IgG-Alexa 488: https://www.abclonal.com.cn/catalog/AS035<br>goat anti-mouse IgG-TRITC antibodie: https://www.abclonal.com.cn/catalog/AS026
